# The Zinc-Schiff Base-Novicidin Complex as a Potential Prostate Cancer Therapy

**DOI:** 10.1371/journal.pone.0163983

**Published:** 2016-10-11

**Authors:** Vedran Milosavljevic, Yazan Haddad, Miguel Angel Merlos Rodrigo, Amitava Moulick, Hana Polanska, David Hynek, Zbynek Heger, Pavel Kopel, Vojtech Adam

**Affiliations:** 1 Department of Chemistry and Biochemistry, Mendel University in Brno, Zemedelska 1, CZ-613 00, Brno, Czech Republic, European Union; 2 Central European Institute of Technology, Brno University of Technology, Purkynova 123, CZ-612 00, Brno, Czech Republic, European Union; 3 Department of Physiology, Faculty of Medicine, Masaryk University, Kamenice 5, CZ-625 00, Brno, Czech Republic, European Union; University of South Alabama Mitchell Cancer Institute, UNITED STATES

## Abstract

Prostate cancer cells control energy metabolism by chelating intracellular zinc. Thus, zinc delivery has been a popular therapeutic approach for prostate cancer. Here, we propose the use of the membrane-penetrating peptide Novicidin connected to zinc-Schiff base as a carrier vehicle for the delivery of zinc to prostate cells. Mass spectrometry, electrochemistry and spectrophotometry confirmed the formation/stability of this complex and provided insight regarding the availability of zinc for complex interactions. This delivery system showed minor toxicity in normal PNT1A cells and high potency towards PC3 tumor cells. The complex preferentially penetrated PC3 tumor cells in contrast to confinement to the membranes of PNT1A. Furthermore, zinc uptake was confirmed in both cell lines. Molecular analysis was used to confirm the activation of zinc stress (e.g., *ZnT-1*) and apoptosis (e.g., *CASP-1*). Our results strongly suggest that the zinc-Schiff base-Novicidin complex has great potential as a novel anticancer drug.

## Introduction

Prostate cancer (PCa) is the most common malignant tumor in males in the developed world, and its prevalence is steadily increasing [1]. PCa that is detected in early stages can be treated with curative treatment modalities, including surgical and radiation therapy. However, the hormonal ablation of metastatic cells can lead to a loss of androgen dependency of PCa cells and thus, a hormone-independent tumor with a very low survival rate [2].

Glandular epithelial cells of the human prostate gland can accumulate a high level of zinc, two- to five-fold greater than the zinc level found in other tissues [3]. Zinc is an essential element required for a variety of enzymes and cellular activities, such as cell growth [4]. Zinc accumulation inhibits mitochondrial aconitase activity, leading to an inability of cells to oxidize citrate [5]. Prostate cells can accumulate high levels of citrate to chelate intracellular zinc, whereas in other cells, 95% of zinc is mostly bound to macromolecules in the immobile form of metalloproteins (such as metallothioneins; MTs) and nucleic acids, among others. Prostate cells acquire zinc through transporter proteins (e.g., ZIP1, ZnT-1) that take up zinc from the extracellular fluid or intracellular vesicles [6,7,8]. Malignant cells rely on low zinc levels to activate the citrate oxidation process, thereby becoming more energy efficient through the completion of the Krebs cycle to produce coupled energy (total energy ~38 ATP), whereas normal epithelial cell accumulation of zinc inhibits the m-aconitase, interrupting the Krebs cycle and eliminating coupled energy (total energy ~14 ATP) [9,10]. Hypothetically, increasing the level of circulating zinc available for cellular uptake could be an efficacious approach for restoring the zinc pathway in malignant cells and thus promote tumor suppression.

The challenge associated with zinc deficiency in malignant cells can be addressed through the use of various delivery systems. In the search for new delivery systems, cell-penetrating peptides (CPP) have attracted attention due to their ability to deliver intracellularly a wide range of molecules [11]. CPP are short peptides of fewer than 30 amino acids that are mostly enriched with positively charged amino acids (e.g., Arg, Lys and His), allowing penetration through the cell membrane via various mechanisms, including endocytosis. CPP can provide the easy delivery of various cell-impermeable covalently or non-covalently conjugated cargos [12,13]. Novicidin (NVC) peptide is a promising delivery system candidate due to several properties, such as its cell-penetrating abilities, a low hemolytic effect, a highly amphipathic alpha helix structure and a high affinity for anionic lipids that are characteristic of cancer cells [14].

**Fig 1:**
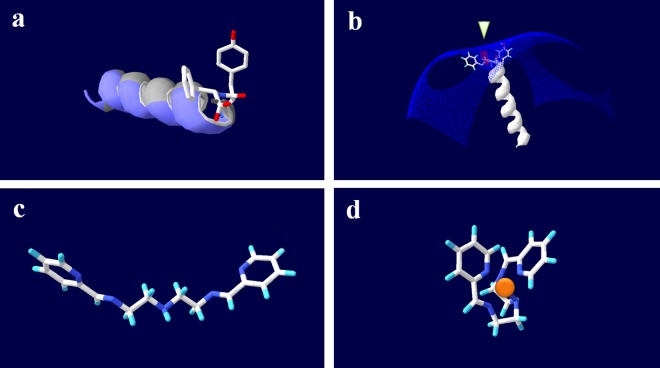
Molecular structure compatibility of Novicidin (NVC) and Schiff base. A) Helix NVC structure prediction using SWISS-MODEL based on the template PDB-ID: 4TQL (Biasini et al. 2014) showing the aromatic ring side chains of the *C*-terminal Tyr-Phe-COO^-^ on a helix ribbon. Positively charged amino acids on the ribbon are shown in blue, while neutral amino acids are shown in gray. After energy minimization (total -900 kJ·mol^-1^), the distance between the aromatic rings was 5.4–8.9 Å. The distance between the aromatic rings and the charged center was 3.4–7.0 Å. B) Electrostatic potential of NVC calculated using DeepView v 4.1 (Guex and Peitsch 1997) and showing the *C*-terminus as the only “negative pocket” in the structure (arrow), represented by the carboxylic acid group surrounded by two aromatic rings. C) Relaxed Schiff base structure without a zinc ion, drawn using Chemwriter (Metamolecular, LLC, USA). The distance between aromatic rings was ~11.4–15.7 Å. D) Zinc-Schiff base structure drawn using Chemwriter showing a positive zinc atom surrounded by two aromatic rings on nearly perpendicular planes. The five nitrogen atoms resulted in a pentahedral shape (9 edges, 6 triangular faces), allowing maximum accessibility to the zinc ion from two opposing sides (edges ~3.3±0.06 Å compared with smaller edges with an average of ~2.5±0.13 Å). One of these sides opposes one aromatic ring, while the other allows for interactions with both rings. The flexibility and directionality in the zinc-Schiff base structure suggests that zinc can be actively accessed even after complexing with peptide. The distance between aromatic rings was ~2.4–6.0 Å. The distance between aromatic rings and charged center was ~1.8 Å. (atoms: red-oxygen, white-carbon, blue-nitrogen, light-blue-hydrogen).

To carry the metal ion cargo, Schiff bases are organic compounds with versatile biological properties. The type of metal and the complexity of the Schiff base play a major role in the potency of these compounds as chemotherapeutic agents. Schiff bases bound to metal ions interact directly with the cell membrane and cause oxidative stress damage. The binding of the Schiff base-metal ion complex with other compounds can neutralize this oxidative capability and permit delivery within biological systems [15,16]. According to the Lewis acid-base theory, the complex of Schiff base–metal ion–peptide (Zn-S-NVC) should be highly stable (Fig 1); both the Schiff base and peptide work together as electron donors to the metal ion, while the metal ion center stabilizes the entire complex [17,18].

Consequently, we were interested in synthesizing a zinc delivery system to increase zinc availability in PCa cells. Here, we investigated the molecular changes that occur after delivery by studying the genes involved in zinc stress. This delivery system showed minor toxicity in normal PNT1A cells and a high potency towards PC3 tumor cells, as shown by a preference to penetrate PC3 cells in contrast to localized confinement to membranes of PNT1A. Furthermore, zinc uptake was confirmed in both cell lines. Molecular analyses were used to confirm the activation of zinc stress (e.g., *ZnT-1*) and apoptosis (e.g., *CASP-1*) genes. For clarity, protein names were shown in normal fonts and gene names were shown in italics.

## Results

### Characterization of the Zn-S-NVC complex

**Fig 2:**
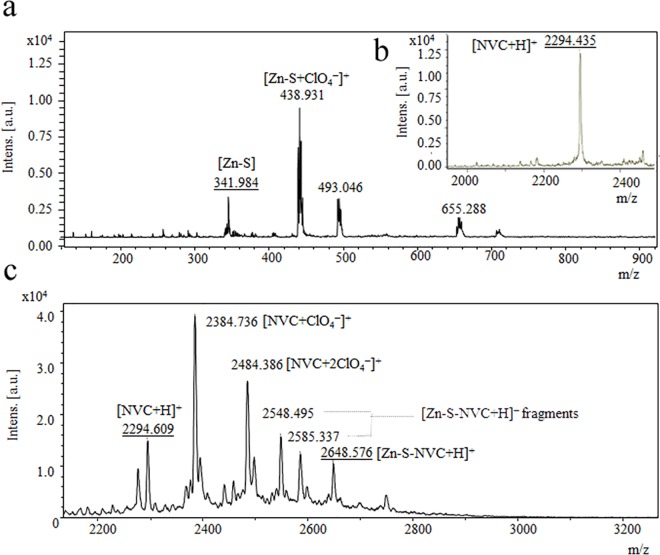
Matrix-assisted laser desorption/ionization time-of-flight mass spectrometry (MALDI-TOF-MS) analysis of the Zinc-Schiff base-Novicidin complex. A) Spectrum of zinc Schiff base [Zn-S]. B) Novicidin peptide [NVC+H]. C) Spectrum of [Zn-S-NVC]. All spectra were measured by MALDI-TOF MS with a 2,5-dihydroxybenzoic acid matrix in TA30 and a maximum energy of 43.2 μJ with a repetition rate of 2000 Hz and 20 subspectra.

As described below, we exploited the interaction of the Schiff base–zinc complex (Zn-S) with the NVC peptide to produce a stable complex for zinc delivery to cells. To confirm this interaction, matrix-assisted laser desorption/ionization time-of-flight mass spectrometry (MALDI-TOF MS) was applied. The observed signals for Zn-S are shown in Fig 2A and were assigned as follows: [Zn-S+H]^+^(*m/z* = 342 Da) corresponds to the ionized Zn-S complex, whereas the peak *m/z* = 439 Da is assigned to the perchlorate adduct of the molecular ion indicating the formation of a hydrogen bond between perchlorate ions and nitrogen donors that have been previously reported for complex ions[19,20]. Fig 2B shows the mass spectrum of ionized NVC [NVC+H]^+^ (*m/z* = 2294 Da). Fig 2C shows the mass spectrum of Zn-S-NVC. The peak at m/z = 2648 Da corresponds to the quasimolecular ion [Zn-S-NVC+H]^+^ and confirms the formation of the Zn-S-NVC complex. In the spectrum, there are also peaks at *m/z* = 2585 Da, 2548 Da, 2484 Da and 2385 Da, which are likely to be fragments of the complex and/or fragments of the complex and organic adduct originating from the matrix, which can be attributed to a high laser intensity and/or the robustness of the crystals that form the DHB matrix. The peak at *m/z* = 2385 Da corresponds to [Zn-NVC+Na]^+^, where the presence of sodium ion can be due to its presence in the matrix, whereas the peaks at *m/z* = 2585 Da and 2484 Da are likely to be perchlorate adducts of [Zn-NVC+Na]^+^. Although we cannot confirm the type of bonding that occurred between the metal and the peptide by MALDI-TOF, complexation between NVC and Zn-S occurred.

**Fig 3:**
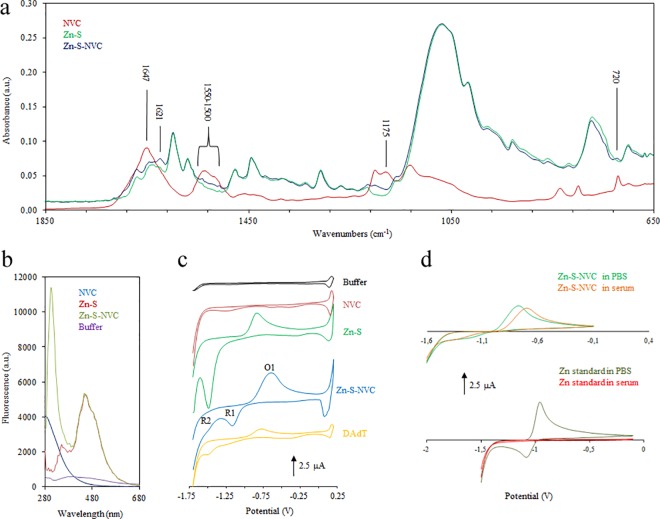
Characterization of Novicidin (NVC), Schiff base (Zn-S) and Zn-S-Novicidin complex (Zn-S-NVC). A) FTIR measurement. B) Fluorescence spectroscopy. C) Real cyclic voltammograms related to the Britton Robinson buffer itself (black line) and prepared complex labeled as follows: NVC (red line), Zn-S (green line), Zn-S-NVC complex determined in the cell (blue line) and Zn-S-NVC complex determined utilizing the double adsorptive transfer technique (orange line). D) Influence of the environment (phosphate buffer and human serum) on the electrochemical response represented by cyclic voltammograms of the free zinc ions (upper part of the figure) and the Zn-S-NVC complex (lower part of the figure). Real cyclic voltammograms were measured in 0.2 M acetate buffer, pH 5.

To better understand the Zn-S interaction with NVC peptide, FTIR measurement was conducted. In the spectrum of Zn-S-NVC, there are slight differences compared with Zn-S, which, as anticipated, occur in the areas where the peptide vibrations are most intense (Fig 3A). The change in the area of 1621 cm^-1^, i.e., near the most intense vibration of free peptide (1647 cm^-1^, which is the area characteristic of the so called amide I., the peak representing the response to the valence vibration C = O in the CONH group) [21]. The shift to lower frequencies could be testified for the possible involvement of atoms from the group interacting with the central zinc atom. Furthermore, as one of the most intense peptide signals in the fingerprint of the Zn-S-NVC spectrum, a signal was apparent at 1175 cm^-1^ that likely represents one of the C = O valence vibrations. The last signal demonstrates the presence of the NVC peptide in the Zn-S-NVC sample at 720 cm^-1^. Due to the intensity of the band in the Zn-S spectrum, it was not possible to reliably identify the remaining peptide vibration in the IR spectrum of Zn-S-NVC; however, it was possible to observe an apparent signal increase in the so-called amide II 1550–1500 cm^-1^ area or even slight differences in the fingerprint area, which could be attributed to the interaction between the peptide and Zn-S. The peaks at 1597 and 1566 cm^-1^ are presented in the spectra of both complexes, and the peaks can be assigned to the ν(C = N) vibration of the Schiff base[22].

Interaction studies between Zn-S and NVC were conducted at room temperature using spectrophotometric methods. The samples were mixed at a 1:1 ratio. The maximum absorbance of all samples was observed at 266 nm. The stability of the formed complex was monitored spectrophotometrically over one week (Figure B in S1 File). An excitation wavelength of 266 nm was used to determine the maximum fluorescence emission wavelength of Zn-S and its interaction with cell-penetrating peptide. However, as shown in Fig 3B, NVC did not show an emission peak in the region of the visible spectrum. The emission spectra of Zn-S showed two peaks at 350 and 450 nm, which are typical for the coordination of Zn ions to Schiff base[23,24]. A comparison of the Zn-S-NVC complex with Zn-S Schiff base revealed that the peak occurred at the same position in both cases, and only the emission of Zn-S-NVC showed one additional peak at 304 nm, which we can assume belongs to the peptide-metal complex representing the Tyr amino acid present in our peptide[25,26].

Furthermore, the interaction of NVC with Zn-S was confirmed and studied using electrochemical methods (Fig 3C). A detailed analysis of data using the double adsorptive transfer technique and cyclic voltammograms is presented in S1 File. The stability of the complex and the environmental influence on Zn-S and NVC was studied in phosphate buffer and human serum. Electrochemical zinc signals (oxidation and reduction) that can be masked in serum were observed in buffer and serum (Fig 3D), suggesting that the complex was stable.

### Cytotoxicity test

**Fig 4:**
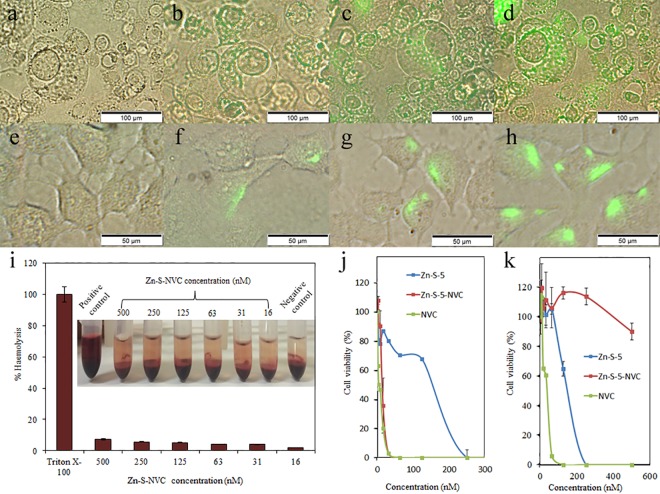
Fluorescence microscopy images. A) PNT1A cells exposed to Zn-S-NVC (conjugated to fluorescent dye) at 0 min. B) PNT1A cells exposed to Zn-S-NVC (conjugated to fluorescent dye) at 30 min. C) PNT1A cells exposed to Zn-S-NVC (conjugated to fluorescent dye) at 60 min. D) PNT1A cells exposed to Zn-S-NVC (conjugated to fluorescent dye) at 90 min. E) PC3 cells exposed to Zn-S-NVC (conjugated to fluorescent dye) at 0 min. F) PC3 cells exposed to Zn-S-NVC (conjugated to fluorescent dye) at 30 min. G) PC3 cells exposed to Zn-S-NVC (conjugated to fluorescent dye) at 60 min. H) PC3 cells exposed to Zn-S-NVC (conjugated to fluorescent dye) at 90 min. I) Haemocompatibility of Zn-S-NVC using human RBCs, showing negligible haemolytic activity in the selected concentration range of Zn-S-NVC (16–500 nM). Inserts show images after incubation and centrifugation. J) MTT analysis of the PC3 cell line. K) MTT analysis of the PNT1A cell line.

We selected the human prostate cell lines PC3 and PNT1A, which are commonly used in biomedical research. Approximately 1000 cells were incubated in 100 μL of a reduced-serum medium for cell culture (Opti-MEM, Life Technologies, USA) under standard conditions (37°C, 5% CO_2_) in an incubator (New Brunswick Eppendorf, USA). The Zn-S-NVC complex was applied to the cells at a final concentration of 100 μM. After different incubation periods (30: 60 and 90 min), the samples were washed three times with PBS buffer to remove the excess of Zn-S-NVC complex. The control sample was maintained under the same conditions without the addition of the Zn-S-NVC complex. PNT1A cells were also prepared using the same protocol for a comparative study of the effect of the Zn-S-NVC complex on cells. Uptake of the Zn-S-NVC complex slowly increased in PC3 cells over time (Fig 4A–4D). Attachment of the Zn-S-NVC complex to PNT1A cells also increased over time (Fig 4E–4H). A comparison of the microscopic images of both cells revealed that the Zn-S-NVC complex was distributed in the cytosol of the PC3 cells, whereas in PNT1A cells, it was confined to the membrane. After 12 h of incubation, the PC3 cells started to exhibit membrane “blebbing,” an indicator of cell death by apoptosis, whereas PNT1A cells remained healthy. This result was later confirmed using the MTT assay.

A haemolytic assay was conducted to study the haemocompatibility of the Zn-S-NVC complex. The haemolytic activity of peptides is a crucial parameter to estimate their therapeutic index. Strong membrane binding and the penetration ability of peptides into cancer cells can also pose a threat to RBC *via* similar membrane-disruption mechanisms. Thus, refining the peptides will decrease these undesired side effects. Fig 4I illustrates that the highest applied concentration of Zn-S-NVC (500 nM) triggered haemolysis of only approximately 7.4% (in relation to Triton X-100 as a positive control). Thus, Zn-S-NVC can be applied with minimal effects on blood circulation.

The cytotoxicity of the Zn-S-NVC complex was examined in PC3 and PNT1A cell lines using the MTT assay. As shown in Fig 4J and 4K, NVC alone induced high toxicity in both PC3 (Fig 4J) and PNT1A (Fig 4K) cell lines, reducing cell viability by 50% at a concentration of 16 nM and by 100% at 63 nM after 24 h. The Zn-S complex without peptide also showed significant toxicity toward both cell lines compared with NVC alone, reducing cell viability by 40% at a concentration of 125 nM and by 100% at 250 nM after 24 h. After the application of the Zn-S-NVC complex to the PC3 cell line (Fig 4J), cell viability was reduced by 65% at a concentration of 46 nM. However, in PNT1A cells, the complex had negligible cytotoxicity, even at a concentration of 500 nM (Fig 4K).

### Expression of genes associated with zinc stress in PCa cells

Human prostate PC3 cancer cells and PNT1A normal cells were incubated with Zn-S-NVC complex at a concentration of 150 μM. Before the treatment with Zn-S-NVC, the concentration of total zinc in cells was determined by AAS as 35.3 μg·mL^-1^. After treatment, the zinc concentration dropped to ~26.4 μg·mL^-1^ in the PC3 culture and to ~27.3 μg·mL^-1^ in the PNT1A culture. Assuming each confluent culture contained 8.4 million cells for both cell lines, the amount of zinc consumed was approximately 5.3 pg per PC3 cell and 4.8 pg per PNT1A cell. Although these numbers are not greatly different from each other, they support the notion that, with the Zn-S-NVC delivery system, PNT1A cells more efficiently exclude zinc than the PC3 cells.

**Fig 5:**
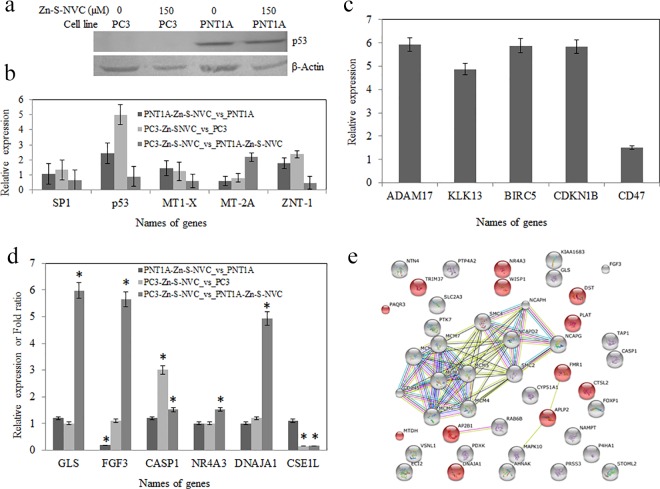
Gene expression analysis. A) Western blot analysis of p53 protein expression revealed no expression in PC3 cells and no change in PNT1A cells. Western blot analysis using anti-p53 (sc-126) after treatment with 150 μM Zn-S-NVC complex for 40 min. Anti-β-Actin (sc-130657) was used as an internal control. B) Change in the gene expression in PC3 and PNT1A cells assessed by qRT-PCR. Relative expression or fold change in genes after treatment with 150 μM Zn-S-NVC complex for 40 min. The qRT-PCR data were normalized to the internal control *18S rRNA*. The mean change is shown for two independent experiments (bars represent the standard deviation). C) Up-regulated genes in PC3 cells before treatment with Zn-S-NVC complex by microarray (PC3_vs_PNT1). D) Relative expression or fold change in genes in response to different treatments after application of Zn-S-NVC complex in PC3 and PNT1A cells by ElectraSense CombiMatrix microarray (bars represent the error standard, * indicates significant differences between treatments). E) Globally up-regulated genes in PC3 and PNT1A cells after treatment with Zn-S-NVC complex assessed using KEGG 10 software (Known and Predicted Protein-Protein Interactions). The red-colored genes indicate genes that negatively regulate cellular processes (metabolic, cell death or apoptotic processes). Different line colors represent the types of evidence for the association (neighborhood, gene fusion, co-occurrence and co-expression).

Several studies have reported changes in the regulation of MT isoforms, zinc transport (ZnT), and tumor protein 53 (p53) in human prostate cells and tissues[8,27,28]. Western blot analysis revealed an absence of p53 protein in PC3 cells. However, there were no visible differences in p53 protein expression in PNT1A cells (Fig 5A). To evaluate the effect of Zn-S-NVC complex delivery on PCa cells, the gene expression in PC3 and PNT1A cells was assessed using quantitative RT-PCR. Three independent experiments were conducted to assess 5 genes after treatment with Zn-S-NVC complex: *SP1*, *p53*, *MT-1X*, *MT-2A* and *ZnT-1*, as shown in Fig 5B.

The expression levels of *p53* increased two-fold in PNT1A cells and five-fold in PC3 cells after treatment. *SP1* transcription factor expression levels remained relatively unchanged after treatment in both normal and tumor cells. Two MT isoforms genes (*MT-1X and MT-2A*) showed variable expression levels, with *MT-2A* expression levels that were 2.2-fold higher in treated PC3 cells compared with treated PNT1A cells. Interestingly, the expression of the zinc transporter *ZnT-1* was increased nearly two-fold after treatment in both PNT1A and PC3 cells.

### Identification of cancer genes expression associated with Zn-S-NVC complex in prostate cell lines by microarray

Further investigation of cancer biomarkers affected by Zn-S-NVC complex treatment was performed. The employed DNA microarray consists of 4×2 k oligonucleotide probes specific to each type of cancer. For this study, we used a commercial chip (Human Cancer 3711 ElectraSense 4×2 k array slides) from CombiMatrix (Custom Array, Bothell, WA, USA) to identify cancer biomarkers that were expressed in PC3 cells treated with Zn-S-NVC complex.

In the present study, we used four different treatments: PNT1A (PNT1A cell line before treatment with Zn-S-NVC complex); PNT1A-Zn-S-NVC (PNT1A cell line after treatment); PC3 (PC3 cell line before treatment); and PC3-Zn-S-NVC (PC3 cell line after treatment). To evaluate the results, we determined the fold ratio or relative expression of genes in all possible combinations between treatments: the effect of Zn-S-NVC complex in PC3 cells (PC3-Zn-S-NVC *vs*. PC3) and in PNT1A cells (PNT1A-Zn-S-NVC *vs*. PNT1A) and the global effect between PC3 and PNT1A cells after treatment with Zn-S-NVC complex (PC3-Zn-S-NVC *vs*. PNT1A-Zn-S-NVC).

The image of the ElectraSense array with a gray scale representing the intensity of expression, number of regulated genes and an overview of regulated genes (up and down-regulation) in the PNT1A and PC3 cell lines in response to the different treatments (after and/or before Zn-S-NVC complex application) are shown in Figure A and Table B in S1 File, respectively.

Our control was the variant PC3 *vs*. PNT1A (before treatment with Zn-S-NVC) to verify the expression of general cancer genes and specific genes related to PCa in our samples (Table B in S1 File). Fig 5C shows the specific genes that were up-regulated in PC3 cells compared with PNT1A cells before treatment with Zn-S-NVC complex, which included *ADAM17*, *KLK13*, *BIRC5*, *CDKN1B*, and *CD47* genes.

Tables C and D in S1 File shows the list of processes and/or pathways of gene regulation in PC3 and PNT1A cells lines comparing different treatments using gene ontology (GO) annotations and KEGG 10 software, respectively. The most important and interesting comparison was the global effect between PC3 and PNT1A cells after treatment with the complex (PC3-Zn-S-NVC *vs*. PNT1A-Zn-S-NVC). Moreover, this comparison revealed the over-expression of different genes from biological pathways related to cell proliferation and cell differentiation (Fig 5D): *GLS* (glutaminase) is a key enzyme that converts glutamine to glutamate and couples glutaminolysis of the tricarboxylic acid cycle (TCA) with elevated glucose uptake and consequently the growth of PCa cells[29]; and *FGF3* (fibroblast growth factor 3) elevates the levels of *FGFRs*, which have been found in numerous human cancers, such as cancers of the brain, head and neck, lung, breast, stomach, and prostate, sarcomas and multiple myeloma[30,31]. Many genes responsible for the negative regulation of cellular metabolic processes and the positive regulation of programmed cell death were up-regulated (*CASP1*, *NR4A3* and *DNAJA1*). These results indicated that genes that induce cell death or apoptosis were activated in PC3 cells after applying the Zn-S-NVC complex (Fig 5E).

## Discussion

Targeting the zinc metabolic pathway to induce apoptosis is a progressive field in PCa therapy. Here, we investigated a novel drug delivery system composed of a membrane-penetrating peptide and a Schiff base as a zinc carrier. Targeting the zinc pathway for PCa treatment has been a research interest of many investigators over the past decade. We believe that developing zinc delivery systems should be the next step for PCa treatment. The versatility of cell-penetrating peptides and Schiff bases will allow development of highly selective and potent therapeutic compounds in the future. In the current study, a NVC conjugate with a Schiff base-zinc complex was synthesized and evaluated for its apoptosis-inducing activity in human PCa cells. This zinc complex potently induced apoptosis in a dose- and time-dependent manner. Our findings reinforce the notion that peptide-carrying zinc compounds have great potential for the development of novel anticancer drugs. This new complex was successfully tested for stability, biocompatibility, cancer specificity, potency and mechanism of action in the PC3 cell line.

The molecular structure of NVC and Schiff base allows a compatible interaction. The NVC peptide is composed of alternating neutral and basic amino acids (Fig 1A). The *C*-terminal carboxylic group forms a negatively charged pocket surrounded by two aromatic rings (Fig 1B). Alternatively, the flexible structure of the Schiff base (Fig 1C) allows the zinc ion to be geometrically coordinated but rather accessible to the NVC carboxylic group from one of two opposing sides (Fig 1D). The two aromatic rings of the Schiff base provide a compatible electron clouds that can interact via π-π stacking with the aromatic rings of Phe and Tyr amino acids and increase the stability of the zinc-Schiff base-NVC complex.

The composition, interactions and stability of the Zn-S-NVC complex was investigated using mass spectrometry (Fig 2), FTIR (Fig 3A), fluorescence spectrometry (Fig 3B), and voltammetric methods (Fig 3C and 3D). The results support the formation of a complex with zinc ion that remains available for interactions within the complex.

Furthermore, we investigated the cytotoxicity of the NVC, Schiff base and delivery system. Our results revealed a decreased toxicity of Zn-S-NVC in the normal PNT1A cell line. A recent study investigating the cytotoxic antimicrobial activity of Melittin and NVC suggested an explanation for the decreased cytotoxicity in some cases [14]. In the presence of low fractions of negatively charged elements in the membrane, the free energy of the interaction between NVC and the membrane increased, leads to decreased cytotoxicity. Similarly, cancer cells display more negatively charged elements on their membrane, thus decreasing the free energy of incorporation and increase the cytotoxicity, as was observed in our experiment [32].

It is unknown whether the Schiff base exerts oxidative stress effects while conjugated to zinc and NVC. Our preliminary analysis using the ratio of gallic acid equivalents per mg of protein (GAE/mg) revealed a change of 113.3% after treatment in PNT1A cells and 125.9% in PC3 [using the Antioxidant Assay Kit (Sigma), and BS-400 Chemistry Analyzer (Mindray Medical International Limited, China)]. This slight increase in gallic acid equivalents supports the notion that oxidative stress is not the main cause of cell death, which indicates that the Schiff base is not the main toxic component of the delivery system.

To study the effects of zinc stress in PNT1A and PC3 cell lines and its role in apoptosis, the expression of several genes was investigated. The transcription factor *SP1* plays a dual role in regulating stress to heavy metals and inducing apoptosis, possibly as a competitor antagonist toward other transcription factors involved in ion stress and apoptosis. Previous studies have reported that overexpression of the transcription factor Sp1 leads to cell cycle inhibition at the G_1_ phase before the onset of apoptosis [33]. SP1 and mutant p53 were found to directly interact, forming a hetero-complex and leading to alterations in downstream target genes of both *SP1* and *p53*. In the case of decreasing *p53* expression due to growth factor deprivation, SP1 is released, leading to apoptosis [34]. It is possible that the SP1-p53 interaction leads to the blockade of DNA binding domains, and thus, p53 and SP1 can be regarded as antagonists [35]. In contrast, SP1 overexpression in tumor cells has been suggested to induce apoptosis by increasing the expression of *p53* and regulating apoptosis-related gene expression. SP1 overexpression also regulates global chromatin condensation and packaging stress that leads to apoptosis [36]. Our results show that the *p53* but not the *SP1* gene was activated by more than two-fold within 40 min of exposure to Zn-S-NVC. PC3 cells carry a mutant *p53* gene with a 1-bp deletion in codon 138 that produces a truncated protein due to the formation of a stop codon at position 169 *via* a frame shift [37], which was undetected by the antibody used herein (clone sc-126). Regarding PNT1A cells, there was no visible change in p53 protein levels after treatment with Zn-S-NVC. Previously, Chuang *et al*. showed that overexpression of the SP1 protein resulted in apoptosis through the overexpression of the p53 protein [36]. This result is consistent with our findings, in which p53 protein expression did not change in PNT1A cells and thus did not lead to apoptosis. Although the *p53* gene was activated at the mRNA level in our experiment, p53 activation at the protein level is a multistep post-translational process resulting in many modifications, in which DNA binding and protein-protein interactions confer stabilization, anti-repression and promoter-specific transcriptional activation [38].

As a part of the dual role of *SP1* in the regulation of heavy metal stress, *SP1* regulates the activity of MT. Li *et al*. showed that *MT-1* is activated by a complex of proteins involving MTF-1, SP1 and p300/CBP [39]. Other reports have shown that SP1 binds to the metallothionein-2A (*MT-2A*) gene and is likely to regulate transcription by competing with the transcriptional activator MTF-1 [40,41]. The MT family of proteins chelates heavy metal ions through their cysteine-rich motifs. Each molecule of MT is capable of binding seven divalent metal ions with variable affinities, with copper having the highest and zinc having the weakest affinity [42]. Our results revealed an increase in expression in one of two MT genes, namely *MT-2A*, upon exposure to Zn-S-NVC complex. MTs are located in the membrane of the Golgi apparatus [43]. Qin *et al*. showed that free zinc ions are mostly concentrated in the cytoplasm (80 pM in HeLa cells) and less concentrated in the endoplasmic reticulum (0.9 pM) and Golgi apparatus (0.6 pM) [44]. One can hypothesize that MTs and other chelating proteins in the ER and Golgi apparatus store zinc and heavy metal ions prior to extracellular secretion. Meplan *et al*. showed that MTs regulate the p53 conformation through their zinc-chelating properties [45]. The p53 DNA binding domain requires zinc for its wild type conformation, and thus, the overexpression of MTs leads to a conformational change in p53 DNA binding domain folding and results in the modulation of p53 transcriptional activity.

However, the regulation of free zinc transport from the cytoplasm to organelles, the nucleus and extracellularly is regulated by specialized zinc transporter proteins. PCa cells accumulate reduced levels of zinc due to a down-regulation of the hormone-responsive zinc transporter *ZIP1* [46]. Normal prostate cells take up zinc either in free or chelated form, e.g., with citrate [47]. A study by Hasumi *et al*. investigating the *ZnT-1* zinc transporter in tumor prostate cell lines showed increased expression of both *ZnT-1* mRNA and protein in the presence of zinc [8]. In the absence of zinc, the authors reported a lower expression level of ZnT-1 in cancer cells compared with normal prostate cells, thus casting more doubt on the role of this protein as a zinc exporter in PCa cells. Our results revealed increased expression levels of *ZnT-1* in both cell lines following treatment. Unfortunately, the regulation of *ZnT-1* remains unclear, e.g., it is unclear whether this gene is hormone-responsive like the *ZIP1* transporter [8]. Cousins and McMahon also noted the variability in *ZnT-1* expression at the mRNA *vs*. the protein level [48]. They studied the role of the energy-independent zinc transporter *ZnT-1* in the detoxification (export) of zinc from cells exposed to high extracellular levels of zinc. Recent reports have also shown discrepancies in the levels of *ZnT-1* in PCa cell lines [49]. Additional studies are required to shed light on the regulation of this zinc transporter and its physiological role in PCa cells. In contrast to our microscopy findings showing peptide confined to the membrane of PNT1A cells, *ZnT-1* expression provided evidence supporting the delivery of zinc in both cell lines. This finding was confirmed by the zinc concentration measured using AAS. It is possible that the peptide component of the complex is only internalized by PC3 cells due to structural differences in their membranes, while Zn-Schiff base is internalized in both PC3 and PNT1A cells.

Microarray was employed to identify cancer biomarkers that are generally over-expressed in the PC3 cell line. The use of this method and its respective apparatus has been previously reported [50]. However, the obtained data do not provide information regarding what happens during cellular metabolism because the chip only identifies general and specific cancer biomarker that are regulated in human samples, i.e., prostate, gastric, lung and brain. Here, we reported the over-expression of several genes: *ADAM17*, *KLK13*, *BIRC5*, *CDKN1B* (p27Kip1) and *CD47*. *ADAM17* over-expression promotes PCa cell proliferation by activating the EGFR/PI3K/AKT pathway [51]. Lose *et al*. showed that the regulation of *KLK13* appears to be more complex in PCa cells depending on the tissue, tumor or cell line [52]. *BIRC5* up-regulation encodes negative regulatory proteins that prevent apoptotic cell death in the lung, pancreas, colon, breast, and PCa [53]. Different studies have suggested that the up-regulation of *CDKN1B* is a prognostic marker in PCa [54]. CD47 is a transmembrane protein that is encoded by the *CD47* gene in humans and is up-regulated in the PC3 cell line. The expression of *CD47* is a general mechanism used by which human solid tumor cells evade phagocytosis. *CD47* expression has been detected on most PCa cells and other cancer cells from primary and xenografted patient tumor samples [55]. Therefore, these results (Fig 5C) verified the expression of specific PCa genes in our samples.

The microarrays results could shed some light on the genes and biomarkers involved in inducing cellular death in PC3 cells after applying the Zn-S-NVC complex. Normal prostate cells accumulate zinc and contain high levels of cellular zinc. In contrast, PCa cells have lost the ability to accumulate zinc and contain low zinc concentrations. Zinc has been reported to induce apoptosis in many mammalian cell types, including prostate epithelial cells [56]. The influence of zinc on apoptosis is a well-known phenomenon [57]. However, *CASP1* overexpression has been shown to induce apoptosis in mammalian cells. *CASP1* is involved in the signal transduction pathways underlying apoptosis, necrosis and inflammation [58]. *CASP1* is a caspase initiator that was originally characterized as cleaving inactive prointerleukin-1 to generate the active proinflammatory cytokine interleukin-1 [59]. Truong-Tran *et al*. examined the cytoprotective functions of zinc, which suppresses the major pathways leading to apoptosis, as well as the more direct influence of zinc on apoptotic regulators, particularly the CASP family of enzymes [60]. In our study, in comparing PC3 cells treated with the Zn-S-NVC complex (PC3-Zn-S-NVC *vs*. PC3) and in comparing the global effect between PC3 and PNT1A after treatment with the Zn-S-NVC complex (PC3-Zn-S-NVC *vs*. PNT1A-Zn-S-NVC), a clear suppression of proliferation was observed. These results may be due to an increase in *CASP1* gene expression, as shown in the Fig 5D. The up-regulation of *CASP1* can be induced to increase intracellular zinc in PC3 cells after treatment with the Zn-S-NVC complex. *CASP1* activation during apoptosis is an important underlying theme in PCa therapy and in recent therapeutic strategies aimed at specifically targeting these proteases in relation to PCa. In addition, the *NR4A3* gene was up-regulated in the global effect between PC3 and PNT1A cell lines after treatment with Zn-S-NVC complex (PC3-Zn-S-NVC *vs*. PNT1A-Zn-S-NVC) (Fig 5D). Neuron-derived orphan receptor 1 (*NOR1*) is a protein that is encoded by the *NR4A3* gene in humans [61]. Shan *et al*. provided the first direct evidence in PC3 that *NOR1* overexpression can lead to apoptosis induction by altering the expression of apoptosis-related genes through the MAPK signaling pathway [62]. Therefore, over-expression of the *NR4A3* gene can be activated by Zn-S-NVC complex in our PC3 cells. Additionally, the microarray results from our samples showed over-expression of the *DNAJA1* gene in the global effect between PC3 and PNT1A cell lines after treatment with Zn-S-NVC (PC3-Zn-S-NVC *vs*. PNT1A-Zn-S-NVC). The *DNAJA1* gene plays an important role in programmed cell death. Stark *et al*. showed that *DNAJA1* overexpression reduces PCa cell survivability under stress, and the J-domain of *DNAJA1* itself may be a valuable biological target for the treatment of PCa as part of a combination therapy [63]. Prior results had not shown the *DNAJA1* gene in the PC3 cell line. Therefore, we report the first information regarding the over-expression of this gene in PC3 cells after treatment with Zn-S-NVC complex. We also can assume that this gene induces apoptosis in PCa cells. However, the *CSE1L* (cellular apoptosis susceptibility protein) gene was down-regulated in response to all treatments in PC3 cells after treatments with Zn-S-NVC (PC3-Zn-S-NVC *vs*. PC3 and PC3-Zn-S-NVC *vs*. PNT1A-Zn-S-NVC) (Fig 5D). Zhu *et al*. showed that *CSE1L* expression was significantly inhibited in a human colon cancer cell line, causing a delay in cell proliferation and induction of apoptosis [64]. Therefore, we can suggest that this down-regulation of *CSE1L* by the Zn-S-NVC complex may be a potential therapeutic approach for PCa.

All presented data strongly suggest that Zn-S-NVC complex can be used as a potent inducer of apoptosis in PCa cells, revealing great potential for its development as a novel anticancer therapy.

## Methods

### Chemicals and reagents

Chemicals and solvents were supplied by Sigma-Aldrich (St. Louis, MO, USA) with ACS purity and used without further purification.

### Synthesis of Schiff base (S), Schiff base complex Zn-S, Novicidin peptide and Zn-S-NVC complex

*Schiff base [(2-[(E)-2-pyridylmethyleneamino]-N-[2-[(E)-2-pyridylmethylene-amino]ethyl]ethanamine)]* was prepared by mixing 2-pyridinecarboxaldehyde (1902 μL) and diethylenetriamine (1080 μL) with stirring and heating under reflux in methanol (35 mL) for 6 h. The color turned to orange; after cooling, methanol was added to 50 mL.

Zinc perchlorate hexahydrate (0.372 g) was dissolved in 50 mL of water, and Schiff base S (5 mL) was added with stirring to obtain Zn-S Schiff base complex. The light orange solution was heated at 80°C for 2 h, the solution was then filtered, and water was added to 100 mL.

To synthesize NVC peptide, Liberty Blue peptide synthesizer was used (CEM, Matthews, NC, USA). The sequences and monoisotopic molecular weight of the synthesized peptide were as follows, respectively: KNLRRIIRKGIHIIKKYF and—2296 Da.

A stock solution of NVC peptide (1 mM) was mixed with Zn-S (1 mM) at a 1:1 ratio. The final concentration of the complex in PBS buffer was 1 mM. The sample was incubated for 60 min at 25°C. After incubation, unbound Zn-S was removed using the fluorescein fast protein liquid chromatography (FPLC) system, Biologic DuoFlow (Bio-Rad, Philadelphia, PA, USA).

### Characterization of Zn-S, NVC and Zn-S-NVC complex

Mass spectrometry experiments were performed using a MALDI-TOF MS Bruker Ultraflextreme (Bruker Daltonik GmbH, Bremen, Germany) equipped with a laser operating at a wavelength of 355 nm with an accelerating voltage of 25 kV, cooled with nitrogen, with a maximum energy of 43.2 μJ and a repetition rate of 2000 Hz in linear and positive mode. Data were acquired and processed using mass spectra flexControl version 3.4 and flexAnalysis version 2.2 software. The 2,5-dihydroxybenzoic acid matrix (DHB) was used for MALDI-TOF (Bruker Daltonik GmbH).

The interactions between Zn-S and NVC were determined using the multifunctional microplate reader, Tecan Infinite 200 PRO (Tecan, Maennedorf, Switzerland). The absorbance spectra were measured within the range from 230–850 nm. For the fluorescence spectra, 230 nm was used as the excitation wavelength, and the fluorescence scan was measured within the range from 260–650 nm. The complex stability was determined by spectra recorded within the range from 190–850 nm using quartz cuvettes (1 cm, Hellma, Essex, UK) on a SPECORD 210 spectrophotometer (Analytik Jena, Germany) at 20°C. Spectra were recorded after 0, 1, 3 and 7 days of incubation. The FTIR spectra (4000–400 cm^–1^) were recorded on a Bruker Tensor 27 spectrometer (Bruker Daltonik GmbH) equipped with a platinum-ATR accessory with a diamond crystal.

Electrochemical measurements were performed with the AUTOLAB Analyzer (EcoChemie, Netherlands) connected to VA-Stand 663 (Metrohm, Switzerland), using a standard cell with three electrodes. Stability experiments for the Zn-S-NVC complex were performed using cyclic voltammetry in acetate buffer (0.2 M CH_3_COONa + CH_3_COOH, pH 5).

### Cultivation of prostatic cell lines

Two human prostatic cell lines were used in this study: the PNT1A human cell line and the PC3 human cell line were purchased from Health Protection Agency Culture Collections (Salisbury, UK). PNT1A cells were cultured in RPMI-1640 with 10% FBS. PC3 cells were cultured in Ham's F12 medium with 7% FBS. All media were supplemented with penicillin (100 U/mL) and streptomycin (0.1 mg/mL), and the cells were maintained at 37°C in a humidified incubator (Sanyo, Moriguchi, Japan) with 5% CO_2_. The total cell content was analyzed using the Casy model TT system (Roche Applied Science, Penzberg, Germany). To prepare a viable cell standard, a 100-μL cell suspension was mixed with 10 mL of Casy Tone. All subsequent measurements were performed with a 100-μL cell suspension diluted 100×. The background was subtracted prior to each measurement.

### Determination of complex cytotoxicity—MTT assay

The suspension of approximately 5000 cells was added to each well of microtiter plates (E-plates 16). Cultures were incubated for 2 days at 37°C to ensure cell growth. The medium was replaced with medium containing Zn-S-NVC complex (2–500 nM), and medium without agents served as a control. The plates were incubated for 24 h, and the medium was replaced with fresh medium three times daily. Additionally, the medium was replaced with 200 μL of fresh medium containing 50 μL of 3-(4,5-dimethylthiazol-2-yl)-2,5-diphenyltetrazolium bromide (MTT [5 mg·mL^-1^ in PBS]) and incubated for 4 h at 37°C. The MTT-containing medium was replaced with 200 μL of 99.9% dimethyl sulfoxide to dissolve MTT-formazan crystals. Then, 25 μL of glycine buffer was added to all wells, and the absorbance was determined at 570 nm (VersaMax microplate reader, Molecular Devices, Sunnyvale, CA, USA). Six replicates were used to assess the Zn-S-NVC complex, and four replicates were used for Zn-S.

### Hemolysis evaluation

Human blood was obtained from the Department of Physiology, Faculty of Medicine, Masaryk University, Czech Republic. We analysed the samples of healthy volunteers (n = 2), whereas the written consent of blood donors was granted. The research has been approved by the Independent ethics committee at University Hospital, Brno, Czech Republic. Red blood cells (RBCs) were obtained from whole blood according to Evans *et al*. [65]. RBC suspensions were washed three times with iso-osmotic PBS (pH 7.4) and then diluted. Five hundred microliters of erythrocyte suspension was interacted with Zn-S-NVC complex at various doses and then incubated for 1 h at 37°C. The degree of hemolysis was determined by measuring the absorbance of the supernatant at 540 nm after centrifugation and calculated according to the following equation: %*hemolysis* = [(*A*_*t*_-*A*_*c*_)/*A*_100%_-*A*_*c*_)]×100, where *A*_*t*_ is the absorbance of the supernatant from samples incubated with Zn-S-NVC; *A*_*c*_ is the absorbance of the supernatant from the negative control (PBS, pH 7.4); *A*_*100%*_ is the absorbance of the supernatant from the positive control (0.1% Triton X-100), which causes complete RBC lysis.

### Microscopy of complexes in ambient light

Zinc-Schiff base-Novicidin complex was conjugated to fluorescent dye [5(6)-carboxyfluorescein *N*-hydroxysuccinimide ester] and purified by FPLC prior to microscopy. An inverted microscope system, Olympus UIS2 series (Tokyo, Japan), was used to image complex internalization into the cells. The CPlanFLN 10× objective was used for 100× magnification, and the LUCPlanFLN 40× objective was used for 400× magnification. The images were captured using a Camera Olympus DP73 and processed with Stream Basic 1.7 Software.

### Atomic absorption spectrometry (AAS)

The total content of zinc in the samples was determined using a 280Z Agilent Technologies atomic absorption spectrometer (Agilent, USA) with electro-thermal atomization. An arsenic ultrasensitive hollow cathode lamp (Agilent) was used as the radiation source (lamp current 10 mA). The spectrometer was operated at a resonance of 193.7 nm with a spectral bandwidth of 0.5 nm.

### RNA extraction and quantitative RT-PCR

The medium was discarded, and the cells were washed with 2 mL of PBS. Approximately 2 mL of either PBS or PBS/Zn-S-NVC was added for both types of cell lines to a final concentration of 150 μM Zn-S-NVC complex. The four cultures were incubated for 40 min with occasional shaking. RNA was isolated using the RNeasy Mini Kit (Qiagen, Venlo, Netherlands).

Gene expression was studied by quantitative RT-PCR using the SYBR Green Quantitative RT-PCR Kit (Sigma-Aldrich, USA) and the Mastercycler pro S instrument (Eppendorf, Hamburg, Germany). The primer sets used to amplify the coding regions of different genes are shown in Table A in S1 File. Each 20-μL reaction contained 2 μL of RNA and a final concentration of 2 μM of each primer. The PCR program consisted of an RT step (44°C/30 min), followed by (94°C/2 min), 35 cycles of (94°C/15 s, 60°C/1 min) and a final extension (68°C/7 min). The specificity of the PCR product was checked by melting curve analysis and 1% agarose gel electrophoresis. The results obtained in triplicates were standardized to the *18S ribosomal RNA* (GenBank Accession X03205.1). The relative levels of transcription were calculated using the 2^−ΔΔCT^ method.

### Protein extraction, SDS-PAGE and Western blotting

Proteins were lysed directly in culture plates with 2 mL of lysis buffer (150 mM NaCl, 1.0% Triton X-100, 50 mM Tris, pH 8.0, and 10 μL of protease inhibitor cocktail (Sigma-Aldrich, USA)) for 30 min. Proteins were electrophoresed in 12.5% polyacrylamide gel at 200 V for 33 min using a Mini Protean Tetra apparatus (Bio-Rad, USA). For Western blotting, PVDF membranes and blotting buffer (25 mM Tris-base, 150 mM glycine and 10% (*v/v*) methanol) were used. The transfer was performed for 1 h at a voltage of 0.9 mA per 1 cm^2^ of the membrane. Anti-β-Actin (sc-130657) and anti-p53 (sc-126) primary antibodies were purchased from Santa Cruz Biotechnology (Dallas, TX, USA) and diluted 1:200. Secondary antibodies were diluted 1:1000. The solution used for calorimetric detection for 1 membrane consisted of 5 mL of 0.01 M acetate buffer pH 5.4, 5 μL of hydrogen peroxide and 50 μL of 3-Amino-9-ethyl-carbazole in DMF.

### Microarray analyses

The cDNA was obtained from 1 μg of total DNase-treated RNA in a 20-μL reaction volume containing 200 units of Super-Script II Reverse Transcriptase (Invitrogen, Carlsbad, CA, USA) and 100 ng of random hexamers. The obtained cDNA was biotinylated at its 3’ end using the Biotin 3’ End DNA labeling kit (Thermo Scientific, Waltham, MA, USA). Microarray was conducted according to Roth *et al*. [66]. For hybridization, Human Cancer 3711 ElectraSense 4×2k array slides were utilized with 1609 DNA probes (Custom Array, Bothell, WA, USA). The detection kit employed for hybridization was CombiMatrix ElectraSense™ and contained Biotin Blocking Solution, Biotin Wash Solution, 3, 3′, 5, 5′-tetramethylbenzidine (TMB) Rinse, and TMB Substrate (CombiMatrix Corporation, USA). The ElectraSense™ application software was used to create a microarray image in gray scale for visualization purposes (Figure A and Table B in S1 File). The list of processes and pathways (Tables C and D in S1 File) of gene regulation in PC3 and PNT1A cell lines showing the comparisons between different treatments was created by gene ontology (GO) annotations and KEGG (Known and Predicted Protein-Protein Interactions) 10 software, respectively.

## Supporting Information

S1 File **Fig A. ElectraSense Array Image. Graphic showing the number of genes regulated in the PC3 and PNT1A cell lines in response to different treatments**. PNT1A (PNT1A cell line before treatment with Zn-S-NVC complex), PNT1A-Zn-S-NVC (PNT1A cell line after treatment with Zn-S-NVC complex), PC3 (PC3 cell line before treatment with Zn-S-NVC complex) and PC3-Zn-S-NVC (PC3 cell line after treatment with Zn-S-5-NVC complex). **Fig B. Stability testing of Zinc-Schiff base-Novicidin complex over one week under different pH conditions.** A) pH = 3.8. B) pH = 6. C) pH = 7.2. D) pH = 9. **Table A. Primers used for quantitative RT-PCR. Table B. Lists of up- and/or down-regulated genes in all possible combinations between treatments:** A) PC3 *vs*. PNT1A. B) PNT1A-Zn-S-NVC *vs*. PNT1A. C) PC3-Zn-S-NVC *vs*. PC3. D) PC3-Zn-S-NVC *vs*. PNT1A-Zn-S-NVC. **Table C. Lists of up- and/or down-regulated genes in biological processes in PC3 and PNT1A cell lines after treatment with Zn-S-NVC complex by gene ontology (GO) annotations.** A) PC3 *vs*. PNT1A. B) PNT1A-Zn-S-NVC *vs*. PNT1A. C) PC3-Zn-S-NVC *vs*. PC3. D) PC3-Zn-S-NVC *vs*. PNT1A-Zn-S-NVC. **Table D. Lists of up- and/or down-regulated genes in various pathways in PC3 and PNT1A cell lines after treatment with Zn-S-NVC complex by KEGG 10 software.** A) PC3 *vs*. PNT1A. B) PNT1A-Zn-S-NVC *vs*. PNT1A. C) PC3-Zn-S-NVC *vs*. PC3. D) PC3-Zn-S-NVC *vs*. PNT1A-Zn-S-NVC.
(DOCX)
