# Correction: The Zinc-Schiff Base-Novicidin Complex as a Potential Prostate Cancer Therapy

**DOI:** 10.1371/journal.pone.0204441

**Published:** 2018-09-17

**Authors:** Vedran Milosavljevic, Yazan Haddad, Miguel Angel Merlos Rodrigo, Amitava Moulick, Hana Polanska, David Hynek, Zbynek Heger, Pavel Kopel, Vojtech Adam

There are a number of errors in the caption for Fig 4, “Fluorescence microscopy images,” panels A-H. Please see the complete, correct Fig 4 caption here.

**Fig 4:**
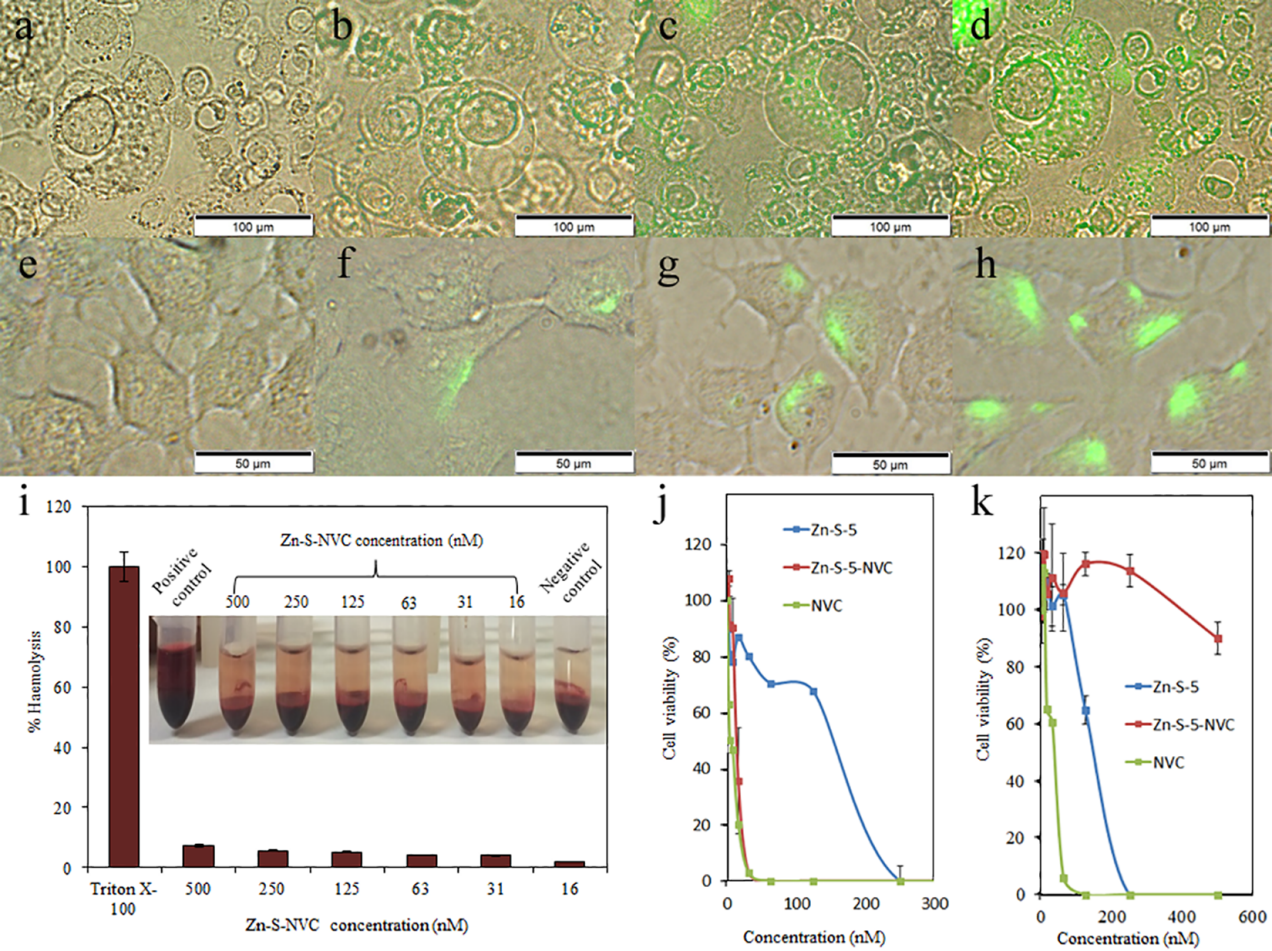
Fluorescence microscopy images. A) PC3 cells exposed to Zn-S-NVC (conjugated to fluorescent dye) at 0 min. B) PC3 cells exposed to Zn-S-NVC (conjugated to fluorescent dye) at 30 min. C) PC3 cells exposed to Zn-S-NVC (conjugated to fluorescent dye) at 60 min. D) PC3 cells exposed to Zn-S-NVC (conjugated to fluorescent dye) at 90 min. E) PNT1A cells exposed to Zn-S-NVC (conjugated to fluorescent dye) at 0 min. F) PNT1A cells exposed to Zn-S-NVC (conjugated to fluorescent dye) at 30 min. G) PNT1A cells exposed to Zn-S-NVC (conjugated to fluorescent dye) at 60 min. H) PNT1A cells exposed to Zn-S-NVC (conjugated to fluorescent dye) at 90 min. I) Haemocompatibility of Zn-S-NVC using human RBCs, showing negligible haemolytic activity in the selected concentration range of Zn-S-NVC (16–500 nM). Inserts show images after incubation and centrifugation. J) MTT analysis of the PC3 cell line. K) MTT analysis of the PNT1A cell line.
