# Correction: The Zinc-Schiff Base-Novicidin Complex as a Potential Prostate Cancer Therapy

**DOI:** 10.1371/journal.pone.0270734

**Published:** 2022-06-24

**Authors:** Vedran Milosavljevic, Yazan Haddad, Miguel Angel Merlos Rodrigo, Amitava Moulick, Hana Polanska, David Hynek, Zbynek Heger, Pavel Kopel, Vojtech Adam

In the versions of Fig 4 appearing in the original article [1] and the initial Correction [2], the results for PC3 cells exposed to Zn-S-NVC for 90 min (Fig 4D) were inadvertently used to represent the results for PC3 cells exposed to Zn-S-NVC for 0 min (Fig 4A). Furthermore, the panels in Fig 4B–4D present cropped areas of the corresponding raw images.

**Fig 4:**
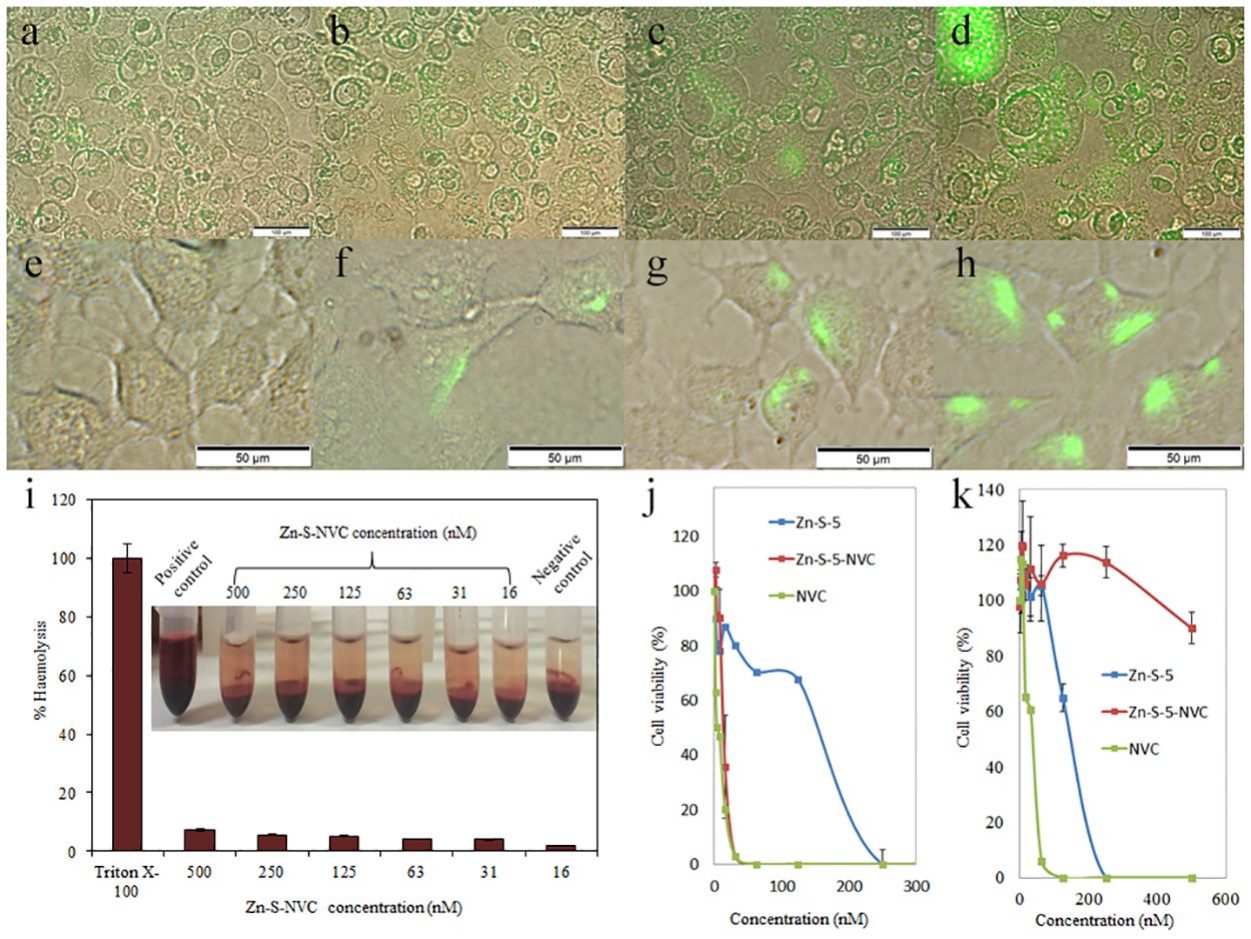
Fluorescence microscopy images. A) PC3 cells exposed to Zn-S-NVC (conjugated to fluorescent dye) at 0 min. B) PC3 cells exposed to Zn-S-NVC (conjugated to fluorescent dye) at 30 min. C) PC3 cells exposed to Zn-S-NVC (conjugated to fluorescent dye) at 60 min. D) PC3 cells exposed to Zn-S-NVC (conjugated to fluorescent dye) at 90 min. E) PNT1A cells exposed to Zn-S-NVC (conjugated to fluorescent dye) at 0 min. F) PNT1A cells exposed to Zn-S-NVC (conjugated to fluorescent dye) at 30 min. G) PNT1A cells exposed to Zn-S-NVC (conjugated to fluorescent dye) at 60 min. H) PNT1A cells exposed to Zn-S-NVC (conjugated to fluorescent dye) at 90 min. I) Haemocompatibility of Zn-S-NVC using human RBCs, showing negligible haemolytic activity in the selected concentration range of Zn-S-NVC (16–500 nM). Inserts show images after incubation and centrifugation. J) MTT analysis of the PC3 cell line. K) MTT analysis of the PNT1A cell line.

An updated version of Fig 4 is provided here containing the correct Fig 4A image. Additionally, for Fig 4B–4D, the full area captured in the corresponding raw images is included.

Raw image data representing brightfield and fluorescence layers for Fig 4A–4D are provided in S1 File. For Fig 4E–4H, high quality raw image data of brightfield and fluorescence layers are no longer available, and lower quality merged images are provided (S1 File).

The Data Availability statement for this paper is incorrect. The correct statement is: Raw data underlying the results reported in this article are available from the corresponding author on request, excluding Fig 4E–4H for which raw image data of brightfield and fluorescence layers are no longer available.

In addition to the above correction of Fig 4, S1 File from [1] is republished here as S2 File with tracking removed.

The corresponding author apologizes for the errors in the published article.

## Supporting information

S1 File Raw image data underlying Fig 4A–4D and merged images corresponding to Fig 4E–4H. (ZIP)

S2 File Updated S1 File from [1]. Fig A. ElectraSense Array Image. Graphic showing the number of genes regulated in the PC3 and PNT1A cell lines in response to different treatments. PNT1A (PNT1A cell line before treatment with Zn-S-NVC complex), PNT1A-Zn-S-NVC (PNT1A cell line after treatment with Zn-S-NVC complex), PC3 (PC3 cell line before treatment with Zn-S-NVC complex) and PC3-Zn-S-NVC (PC3 cell line after treatment with Zn-S-5-NVC complex). Fig B. Stability testing of Zinc-Schiff base-Novicidin complex over one week under different pH conditions. A) pH = 3.8. B) pH = 6. C) pH = 7.2. D) pH = 9. Table A. Primers used for quantitative RT-PCR. Table B. Lists of up- and/or down-regulated genes in all possible combinations between treatments: A) PC3 vs. PNT1A. B) PNT1A-Zn-S-NVC vs. PNT1A. C) PC3-Zn-S-NVC vs. PC3. D) PC3-Zn-S-NVC vs. PNT1A-Zn-S-NVC. Table C. Lists of up- and/or down-regulated genes in biological processes in PC3 and PNT1A cell lines after treatment with Zn-S-NVC complex by gene ontology (GO) annotations. A) PC3 vs. PNT1A. B) PNT1A-Zn-S-NVC vs. PNT1A. C) PC3-Zn-S-NVC vs. PC3. D) PC3-Zn-S-NVC vs. PNT1A-Zn-S-NVC. Table D. Lists of up- and/or down-regulated genes in various pathways in PC3 and PNT1A cell lines after treatment with Zn-S-NVC complex by KEGG 10 software. A) PC3 vs. PNT1A. B) PNT1A-Zn-S-NVC vs. PNT1A. C) PC3-Zn-S-NVC vs. PC3. D) PC3-Zn-S-NVC vs. PNT1A-Zn-S-NVC.
(DOCX)
