# Supplementary material for: The Zinc-Schiff Base-Novicidin Complex as a Potential Prostate Cancer Therapy
Source: PLoS One. 2016 Oct 11;11(10):e0163983. doi: 10.1371/journal.pone.0163983 (PMC5058503; doi:10.1371/journal.pone.0163983)
Supplement: S1 File — Fig A. ElectraSense Array Image. Graphic showing the number of genes regulated in the PC3 and PNT1A cell lines in response to different treatments. PNT1A (PNT1A cell line before treatment with Zn-S-NVC complex), PNT1A-Zn-S-NVC (PNT1A cell line after treatment with Zn-S-NVC complex), PC3 (PC3 cell line before treatment with Zn-S-NVC complex) and PC3-Zn-S-NVC (PC3 cell line after treatment with Zn-S-5-NVC complex). Fig B. Stability testing of Zinc-Schiff base-Novicidin complex over one week under different pH conditions. A) pH = 3.8. B) pH = 6. C) pH = 7.2. D) pH = 9. Table A. Primers used for quantitative RT-PCR. Table B. Lists of up- and/or down-regulated genes in all possible combinations between treatments: A) PC3 vs. PNT1A. B) PNT1A-Zn-S-NVC vs. PNT1A. C) PC3-Zn-S-NVC vs. PC3. D) PC3-Zn-S-NVC vs. PNT1A-Zn-S-NVC. Table C. Lists of up- and/or down-regulated genes in biological processes in PC3 and PNT1A cell lines after treatment with Zn-S-NVC complex by gene ontology (GO) annotations. A) PC3 vs. PNT1A. B) PNT1A-Zn-S-NVC vs. PNT1A. C) PC3-Zn-S-NVC vs. PC3. D) PC3-Zn-S-NVC vs. PNT1A-Zn-S-NVC. Table D. Lists of up- and/or down-regulated genes in various pathways in PC3 and PNT1A cell lines after treatment with Zn-S-NVC complex by KEGG 10 software. A) PC3 vs. PNT1A. B) PNT1A-Zn-S-NVC vs. PNT1A. C) PC3-Zn-S-NVC vs. PC3. D) PC3-Zn-S-NVC vs. PNT1A-Zn-S-NVC. (DOCX) [file pone.0163983.s001.docx]

|  | **Table of Content** | |
| --- | --- | --- |
|  |  |  |
| **Subject** |  | **Page** |
| Supplementary Figure A |  | 2 |
| Supplementary Figure B |  | 3 |
| Supplementary Table A |  | 4 |
| Supplementary Table B |  | 5 |
| Supplementary Table C |  | 13 |
| Supplementary Table D |  | 19 |
| Supplementary Note S1 |  | 22 |

| 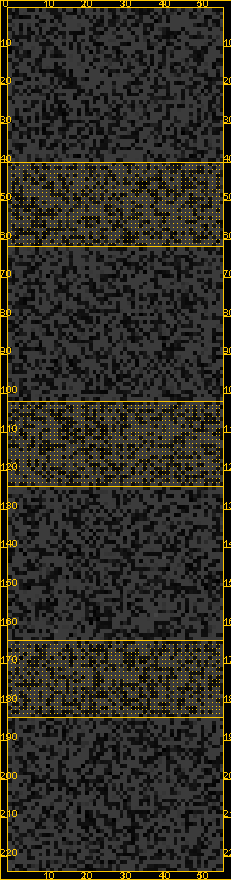  PNT1-Zn-S-NVC | PC3  Inter-place. No hybridation zone  PC3-Zn-S-NVC  Inter-place. No hybridation zone  Inter-place. No hybridation zone  PNT1 |  |  |  |  |  |  |  |
| --- | --- | --- | --- | --- | --- | --- | --- | --- |

**Supplementary Fig A**. **Image of Electrosense Array. Graphic of number of genes of regulation in PC3 and PNT1A cell lines in different treatments.** PNT1A (PNT1A cell line before treated with Zn-S-NVC complex), PNT1A-Zn-S-NVC (PNT1A cell line after treated with Zn-S-NVC complex), PC3 (PC3 cell line before treated with Zn-S-NVC complex) and PC3-Zn-S-NVC (PC3 cell line after treated with Zn-S-5-NVC complex).


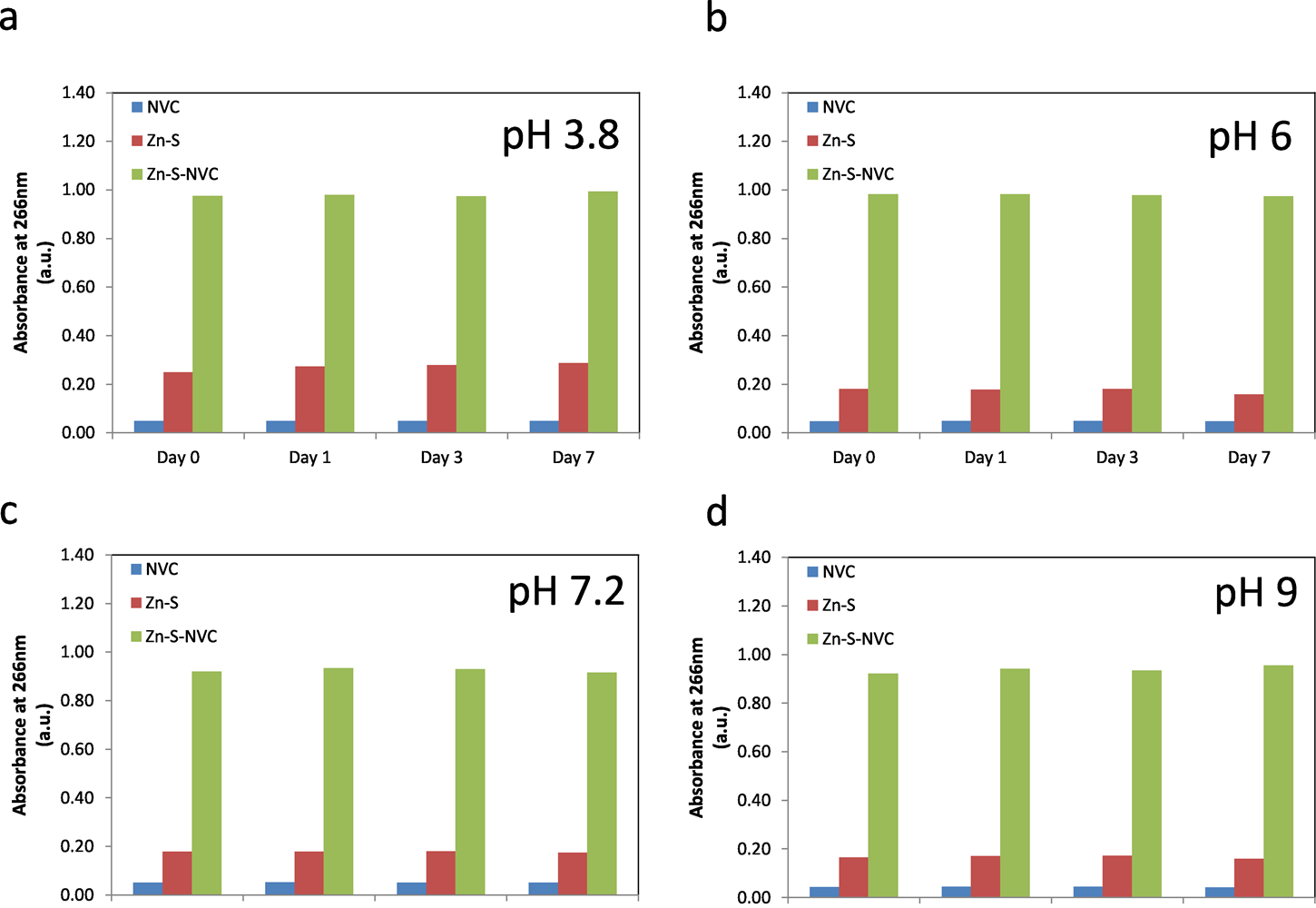


**Supplementary Fig B. Stability testing of Zinc-Schiff base-Novicidin complex over one week under different pH**

**conditions.** a) pH = 3.8. b) pH = 6. c) pH = 7.2. d) pH = 9.

**Supplementary Table A.** Primers used for quantitative RT-PCR

| Gene | Accession  ID | Forward primer | Reverse primer | Product size  (bp) |
| --- | --- | --- | --- | --- |
| *SP1* | NM_138473.2 | CCACCATGAGCGACCAAGAT | AAGGCACCACCACCATTACC | 109 |
| *p53* | NM_000546.5 | TAACAGTTCCTGCATGGGCGGC | AGGACAGGCACAAACACGCACC | 121 |
| *MT-1X* | NM_005952.3 | TCTCCTTGCCTCGAAATGGAC | GGGCACACTTGGCACAGC | 151 |
| *MT-2A* | NM_005953.3 | CCGACTCTAGCCGCCTCTT | GTGGAAGTCGCGTTCTTTACA | 259 |
| *ZnT-1* | NM_021194.2 | AATACCAGCAACTCCAACGG | ATCACTGAACCCAAGGCATC | 200 |
| *18S rRNA* | X03205.1 | CGGCTACCACATCCAAGGAA | GCTGGAATTACCGCGGCTGC | 187 |

Supplementary Table B. Lists of genes up and/or down regulation in all possible combination between treatments: A) PC3 vs PNT1A. B) PNT1A-Zn-S-NVC vs PNT1A. C) PC3-Zn-S-NVC vs PC3. D) PC3-Zn-S-NVC vs PNT1A-Zn-S-NVC.

| A) PC3 vs PNT1A |  |  |  |  |
| --- | --- | --- | --- | --- |
| UP_REGULATION |  |  |  |  |
| Name of Gene | Symbol | NCBI database | Fold_ratio | Error Standar |
| ADAM metallopeptidase domain 17 (tumor necrosis factor, alpha, converting enzyme) | ADAM17 | NM_003183 | 5,93 | 2,65 |
| 60S ribosomal protein L6 (RPL6A) | NAP1L1 | NM_004537 | 5,91 | 2,64 |
| Tweety homolog 1 (Drosophila) | TTYH1 | NM_001005367 | 5,90 | 2,64 |
| Effector cell peptidase receptor 1 | BIRC5 | NM_001012270 | 5,89 | 2,63 |
| Thyroid hormone receptor, alpha (erythroblastic leukemia viral (v-erb-a) oncogene homolog, avian) | THRA | NM_199334 | 5,87 | 2,63 |
| Cyclin-dependent kinase inhibitor 1B (p27, Kip1) | CDKN1B | NM_004064 | 5,84 | 2,61 |
| RB1-inducible coiled-coil 1 | RB1CC1 | NM_014781 | 5,84 | 2,61 |
| Protective protein for beta-galactosidase (galactosialidosis) | PPGB | NM_000308 | 5,83 | 2,61 |
| 5-hydroxytryptamine (serotonin) receptor 6 | HTR6 | NM_000871 | 5,82 | 2,60 |
| Exostoses (multiple) 1 | EXT1 | NM_000127 | 5,74 | 2,57 |
| Gamma-aminobutyric acid (GABA) B receptor, 1 | GABBR1 | NM_001470 | 5,68 | 2,54 |
| Ribophorin II | RPN2 | NM_002951 | 5,64 | 2,52 |
| Nuclear receptor coactivator 4 | NCOA4 | NM_005437 | 5,63 | 2,52 |
| SRY (sex determining region Y)-box 4 | SOX4 | NM_003107 | 5,62 | 2,51 |
| Interleukin 1 receptor-like 1 | IL1RL1 | NM_003856 | 5,54 | 2,48 |
| ARP2 actin-related protein 2 homolog (yeast) | ACTR2 | NM_001005386 | 5,31 | 2,38 |
| Protein kinase C, beta 1 | PRKCB1 | NM_212535 | 5,29 | 2,37 |
| Poly(A) binding protein, cytoplasmic 1 | PABPC1 | NM_002568 | 5,18 | 2,32 |
| Kallikrein 13 | KLK13 | NM_015596 | 4,87 | 2,18 |
| B-cell CLL/lymphoma 7A | BCL7A | NM_020993 | 4,79 | 2,14 |
| Transcription factor 7 (T-cell specific, HMG-box) | TCF7 | NM_201633 | 4,70 | 2,10 |
| Chromodomain helicase DNA binding protein 1 | CHD1 | NM_001270 | 4,23 | 1,89 |
| Karyopherin alpha 2 (RAG cohort 1, importin alpha 1) | KPNA2 | NM_002266 | 4,12 | 1,84 |
| Chemokine (C-X-C motif) ligand 5 | CXCL5 | NM_002994 | 1,60 | 0,72 |
| ATP-binding cassette, sub-family B (MDR/TAP), member 7 | ABCB7 | NM_004299 | 1,58 | 0,71 |
| DAZ associated protein 2 | DAZAP2 | NM_014764 | 1,57 | 0,70 |
| Lactotransferrin | LTF | NM_002343 | 1,55 | 0,69 |
| Inositol hexaphosphate kinase 2 | IHPK2 | NM_001005909 | 1,55 | 0,69 |
| Nuclear receptor subfamily 4, group A, member 2 | NR4A2 | NM_006186 | 1,55 | 0,69 |
| Small EDRK-rich factor 1A (telomeric) | SERF1A | NM_021967 | 1,55 | 0,69 |
| Proliferating cell nuclear antigen | PCNA | NM_182649 | 1,54 | 0,69 |
| Tropomyosin 4 | TPM4 | NM_003290 | 1,54 | 0,69 |
| Abhydrolase domain containing 2 | ABHD2 | NM_007011 | 1,53 | 0,68 |
| Solute carrier family 6 (neurotransmitter transporter, dopamine), member 3 | SLC6A3 | NM_001044 | 1,53 | 0,68 |
| Fusion (involved in t(12;16) in malignant liposarcoma) | FUS | NM_001010850 | 1,53 | 0,68 |
| Microsomal glutathione S-transferase 3 | MGST3 | NM_004528 | 1,52 | 0,68 |
| Coactivator-associated arginine methyltransferase 1 | CARM1 | NM_199141 | 1,51 | 0,68 |
| BCL2-associated athanogene | BAG1 | NM_004323 | 1,51 | 0,68 |
| Choline kinase alpha | CHKA | NM_001277 | 1,51 | 0,67 |
| Nuclear receptor subfamily 5, group A, member 2 | NR5A2 | NM_003822 | 1,51 | 0,67 |
| Egl nine homolog 1 (C. elegans) | EGLN1 | NM_022051 | 1,51 | 0,67 |
| Chromobox homolog 3 (HP1 gamma homolog, Drosophila) | CBX3 | NM_016587 | 1,51 | 0,67 |
| HMT1 hnRNP methyltransferase-like 2 (S. cerevisiae) | HRMT1L2 | NM_001536 | 1,50 | 0,67 |
| Latent transforming growth factor beta binding protein 2 | LTBP2 | NM_000428 | 1,50 | 0,67 |
| CD47 antigen (Rh-related antigen, integrin-associated signal transducer) | CD47 | NM_001025079 | 1,50 | 0,67 |
| TAR (HIV) RNA binding protein 1 | TARBP1 | NM_005646 | 1,50 | 0,67 |
| DOWN_REGULATION |  |  |  |  |
| Name of Gene | Symbol | NCBI database | Fold_ratio | Error Standar |
| DEP domain containing 1 | DEPDC1 | NM_017779 | 0,29 | 0,01 |
| Outer mitochondrial membrane cytochrome b5 | CYB5-M | NM_030579 | 0,27 | 0,03 |
| Sjogren's syndrome nuclear autoantigen 1 | SSNA1 | NM_003731 | 0,22 | 0,01 |
| Bone morphogenetic protein 1 | BMP1 | NM_006132 | 0,21 | 0,05 |
| Nucleoporin 160kDa | NUP160 | NM_015231 | 0,21 | 0,01 |
| Protease, serine, 1 (trypsin 1) | PRSS1 | NM_002769 | 0,21 | 0,04 |
| Lumican | LUM | NM_002345 | 0,19 | 0,00 |
| RAN binding protein 9 | RANBP9 | NM_005493 | 0,19 | 0,02 |
| Embryonic ectoderm development | EED | NM_003797 | 0,19 | 0,02 |
| Carcinoembryonic antigen-related cell adhesion molecule 7 | CEACAM7 | NM_006890 | 0,19 | 0,01 |
| Nuclear receptor subfamily 0, group B, member 2 | NR0B2 | NM_021969 | 0,18 | 0,02 |
| Integrin, beta 5 | ITGB5 | NM_002213 | 0,18 | 0,03 |
| Poly(rC) binding protein 2 | PCBP2 | NM_031989 | 0,18 | 0,01 |
| Pirin (iron-binding nuclear protein) | PIR | NM_001018109 | 0,18 | 0,00 |
| Transient receptor potential cation channel, subfamily C, member 6 | TRPC6 | NM_004621 | 0,18 | 0,01 |
| Proprotein convertase subtilisin/kexin type 6 | PCSK6 | NM_138323 | 0,18 | 0,03 |
| E2F transcription factor 3 | E2F3 | NM_001949 | 0,18 | 0,02 |

| B) PNT1A-Zn-S-NVC vs PNT1A |  |  |  |  |
| --- | --- | --- | --- | --- |
| UP_REGULATION |  |  |  |  |
| Name of Gene | Symbol | NCBI database | Fold_ratio | Error Standar |
| Protein inhibitor of activated STAT, 1 | PIAS1 | NM_016166 | 7,74 | 0,39 |
| Fructose-1,6-bisphosphatase 1 | FBP1 | NM_000507 | 4,79 | 0,28 |
| MAD2 mitotic arrest deficient-like 1 (yeast) | MAD2L1 | NM_002358 | 1,61 | 0,06 |
| Collagen, type IV, alpha 2 | COL4A2 | NM_001846 | 1,59 | 0,01 |
| Microsomal glutathione S-transferase 3 | MGST3 | NM_004528 | 1,58 | 0,01 |
| Growth differentiation factor 15 | GDF15 | NM_004864 | 1,57 | 0,10 |
| CD47 antigen (Rh-related antigen, integrin-associated signal transducer) | CD47 | NM_001025079 | 1,57 | 0,00 |
| Nuclear receptor subfamily 5, group A, member 2 | NR5A2 | NM_003822 | 1,55 | 0,00 |
| Coactivator-associated arginine methyltransferase 1 | CARM1 | NM_199141 | 1,54 | 0,04 |
| RAS p21 protein activator 3 | RASA3 | NM_007368 | 1,54 | 0,01 |
| Small nuclear ribonucleoprotein polypeptide N | SNRPN | NM_022805 | 1,54 | 0,03 |
| Proprotein convertase subtilisin/kexin type 6 | PCSK6 | NM_138320 | 1,54 | 0,04 |
| Creatine kinase, brain | CKB | NM_001823 | 1,54 | 0,05 |
| Egl nine homolog 1 (C. elegans) | EGLN1 | NM_022051 | 1,53 | 0,03 |
| Choline kinase alpha | CHKA | NM_001277 | 1,53 | 0,01 |
| Lactotransferrin | LTF | NM_002343 | 1,53 | 0,03 |
| ATP-binding cassette, sub-family B (MDR/TAP), member 7 | ABCB7 | NM_004299 | 1,53 | 0,04 |
| DAZ associated protein 2 | DAZAP2 | NM_014764 | 1,53 | 0,03 |
| Microfibrillar-associated protein 2 | MFAP2 | NM_002403 | 1,53 | 0,02 |
| Promyelocytic leukemia | PML | NM_033239 | 1,53 | 0,00 |
| Hydroxyprostaglandin dehydrogenase 15-(NAD) | HPGD | NM_000860 | 1,52 | 0,06 |
| Phosphodiesterase 6C, cGMP-specific, cone, alpha prime | PDE6C | NM_006204 | 1,51 | 0,02 |
| TAR (HIV) RNA binding protein 1 | TARBP1 | NM_005646 | 1,51 | 0,04 |
| Dystonin | DST | NM_015548 | 1,51 | 0,00 |
| Syntaxin 1A (brain) | STX1A | NM_004603 | 1,51 | 0,02 |
| BCL2-associated athanogene | BAG1 | NM_004323 | 1,51 | 0,02 |
| Sema domain, immunoglobulin domain (Ig), short basic domain, secreted, (semaphorin) 3B | SEMA3B | NM_001005914 | 1,51 | 0,00 |
| Abhydrolase domain containing 2 | ABHD2 | NM_007011 | 1,51 | 0,03 |
| Effector cell peptidase receptor 1 | BIRC5 | NM_001012270 | 1,51 | 0,04 |
| Nuclear receptor subfamily 1, group I, member 2 | NR1I2 | NM_003889 | 1,51 | 0,04 |
| Tropomyosin 4 | TPM4 | NM_003290 | 1,51 | 0,04 |
| HMT1 hnRNP methyltransferase-like 2 (S. cerevisiae) | HRMT1L2 | NM_001536 | 1,51 | 0,02 |
| Proliferating cell nuclear antigen | PCNA | NM_182649 | 1,51 | 0,04 |
| Interleukin 1 receptor-like 1 | IL1RL1 | NM_016232 | 1,51 | 0,05 |
| Protein tyrosine phosphatase, receptor type, O | PTPRO | NM_002848 | 1,51 | 0,01 |
| Chromobox homolog 3 (HP1 gamma homolog, Drosophila) | CBX3 | NM_016587 | 1,50 | 0,01 |
| DEP domain containing 1 | DEPDC1 | NM_017779 | 1,50 | 0,05 |
| DOWN_REGULATION |  |  |  |  |
| Name of Gene | Symbol | NCBI database | Fold_ratio | Error Standar |
| PTK7 protein tyrosine kinase 7 | PTK7 | NM_002821 | 0,28 | 0,02 |
| Solute carrier family 2 (facilitated glucose transporter), member 3 | SLC2A3 | NM_006931 | 0,26 | 0,04 |
| Protein tyrosine phosphatase type IVA, member 2 | PTP4A2 | NM_080391 | 0,24 | 0,03 |
| Serum amyloid A2 | SAA1 | NM_199161 | 0,22 | 0,04 |
| Peroxisomal D3,D2-enoyl-CoA isomerase | PECI | NM_006117 | 0,20 | 0,02 |
| SMC4 structural maintenance of chromosomes 4-like 1 (yeast) | SMC4L1 | NM_001002799 | 0,20 | 0,01 |
| Plasminogen activator, tissue | PLAT | NM_000931 | 0,20 | 0,02 |
| Progestin and adipoQ receptor family member III | PAQR3 | NM_177453 | 0,19 | 0,03 |
| Fragile X mental retardation 1 | FMR1 | NM_002024 | 0,19 | 0,01 |
| Visinin-like 1 | VSNL1 | NM_003385 | 0,19 | 0,02 |
| Matrilin 2 | MATN2 | NM_002380 | 0,19 | 0,02 |
| Mitogen-activated protein kinase 10 | MAPK10 | NM_138980 | 0,19 | 0,00 |
| Cathepsin L2 | CTSL2 | NM_001333 | 0,19 | 0,01 |
| Thymidylate synthetase | TYMS | NM_001071 | 0,18 | 0,01 |
| Cadherin 3, type 1, P-cadherin (placental) | CDH3 | NM_001793 | 0,18 | 0,02 |
|  | FGF3 | NM_005247 | 0,18 | 0,02 |

| C) PC3-Zn-S-NVC vs PC3 |  |  |  |  |
| --- | --- | --- | --- | --- |
| UP_REGULATION |  |  |  |  |
| Name of Gene | Symbol | NCBI database | Fold_ratio | Error Standar |
| Procollagen C-endopeptidase enhancer | PCOLCE | NM_002593 | 5,99 | 0,69 |
| Ribosomal protein S26 | RPS26 | NM_001029 | 5,98 | 0,10 |
| Ferritin, light polypeptide | FTL | NM_000146 | 5,97 | 0,20 |
| UDP glucuronosyltransferase 1 family, polypeptide A9 | UGT1A6 | NM_001072 | 5,93 | 0,00 |
| Fms-related tyrosine kinase 1 (vascular endothelial growth factor/vascular permeability factor receptor) | FLT1 | NM_002019 | 5,93 | 0,41 |
| Kallikrein 15 | KLK15 | NM_017509 | 5,91 | 1,08 |
| Fucosyltransferase 8 (alpha (1,6) fucosyltransferase) | FUT8 | NM_004480 | 5,88 | 0,72 |
| Nuclear receptor subfamily 0, group B, member 2 | NR0B2 | NM_021969 | 5,84 | 0,65 |
| F-box protein 31 | FBXO31 | NM_024735 | 5,77 | 0,16 |
| Rho GDP dissociation inhibitor (GDI) beta | ARHGDIB | NM_001175 | 5,77 | 0,09 |
| Outer mitochondrial membrane cytochrome b5 | CYB5-M | NM_030579 | 5,71 | 0,59 |
| Transmembrane protein 45A | FLJ10134 | NM_018004 | 5,71 | 0,45 |
| Pirin (iron-binding nuclear protein) | PIR | NM_001018109 | 5,68 | 0,09 |
| Integrin, beta 5 | ITGB5 | NM_002213 | 5,65 | 1,05 |
| UDP-Gal:betaGlcNAc beta 1,4- galactosyltransferase, polypeptide 1 | B4GALT1 | NM_001497 | 5,63 | 0,31 |
| Interleukin 1 receptor-like 1 | IL1RL1 | NM_016232 | 5,59 | 0,43 |
| Guanine nucleotide binding protein (G protein), alpha z polypeptide | GNAZ | NM_002073 | 5,56 | 0,77 |
| Poly(rC) binding protein 2 | PCBP2 | NM_031989 | 5,50 | 0,50 |
| Sjogren's syndrome nuclear autoantigen 1 | SSNA1 | NM_003731 | 5,46 | 0,11 |
| Heat shock 70kDa protein 9B (mortalin-2) | HSPA9B | NM_004134 | 5,46 | 0,60 |
| DEP domain containing 1 | DEPDC1 | NM_017779 | 5,43 | 0,17 |
| Carcinoembryonic antigen-related cell adhesion molecule 7 | CEACAM7 | NM_006890 | 5,33 | 0,19 |
| Protease, serine, 1 (trypsin 1) | PRSS1 | NM_002769 | 5,16 | 1,11 |
| Lumican | LUM | NM_002345 | 5,09 | 0,02 |
| RAN binding protein 9 | RANBP9 | NM_005493 | 5,02 | 0,34 |
| Glycerol-3-phosphate dehydrogenase 1-like | GPD1L | NM_015141 | 4,44 | 0,18 |
| Embryonic ectoderm development | EED | NM_003797 | 4,02 | 0,35 |
| E2F transcription factor 3 | E2F3 | NM_001949 | 3,90 | 0,58 |
| Protein inhibitor of activated STAT, 1 | PIAS1 | NM_016166 | 3,90 | 0,12 |
| Plasminogen activator, tissue | PLAT | NM_000930 | 1,59 | 0,01 |
| DnaJ (Hsp40) homolog, subfamily B, member 6 | DNAJB6 | NM_058246 | 1,57 | 0,02 |
| Eukaryotic translation initiation factor 2C, 2 | EIF2C2 | NM_012154 | 1,54 | 0,03 |
| Thyroid hormone receptor interactor 13 | TRIP13 | NM_004237 | 1,54 | 0,06 |
| BMP and activin membrane-bound inhibitor homolog (Xenopus laevis) | BAMBI | NM_012342 | 1,53 | 0,00 |
| Amino-terminal enhancer of split | AES | NM_001130 | 1,53 | 0,01 |
| Beta-1,3-glucuronyltransferase 1 (glucuronosyltransferase P) | B3GAT1 | NM_018644 | 1,53 | 0,04 |
| Bone morphogenetic protein 1 | BMP1 | NM_006128 | 1,52 | 0,03 |
| Carcinoembryonic antigen-related cell adhesion molecule 1 (biliary glycoprotein) | CEACAM1 | NM_001024912 | 1,52 | 0,05 |
| GDP dissociation inhibitor 1 | GDI1 | NM_001493 | 1,51 | 0,07 |
| Pyrroline-5-carboxylate reductase 1 | PYCR1 | NM_006907 | 1,51 | 0,03 |
| Proteasome (prosome, macropain) activator subunit 1 (PA28 alpha) | PSME1 | NM_176783 | 1,51 | 0,02 |
| KIAA1683 | KIAA1683 | NM_025249 | 1,51 | 0,02 |
| PTK7 protein tyrosine kinase 7 | PTK7 | NM_002821 | 1,50 | 0,02 |
| Chemokine (C-X-C motif) ligand 14 | CXCL14 | NM_004887 | 1,50 | 0,02 |
| Gamma-aminobutyric acid (GABA) B receptor, 1 | GABBR1 | NM_021904 | 1,50 | 0,02 |
| DOWN_REGULATION |  |  |  |  |
| Name of Gene | Symbol | NCBI database | Fold_ratio | Error Standar |
| Hypothetical protein MAC30 | MAC30 | NM_014573 | 0,25 | 0,04 |
| Small nuclear ribonucleoprotein polypeptides B and B1 | SNRPB | NM_198216 | 0,22 | 0,01 |
| Vanin 1 | VNN1 | NM_004666 | 0,21 | 0,03 |
| Speckle-type POZ protein | SPOP | NM_001007226 | 0,21 | 0,01 |
| Parathyroid hormone-like hormone | PTHLH | NM_198966 | 0,20 | 0,03 |
| Disabled homolog 2, mitogen-responsive phosphoprotein (Drosophila) | DAB2 | NM_001343 | 0,20 | 0,02 |
| Exosome component 9 | EXOSC9 | NM_005033 | 0,19 | 0,05 |
| Calnexin | CANX | NM_001024649 | 0,19 | 0,01 |
| Cell division cycle 2-like 6 (CDK8-like) | CDC2L6 | NM_015076 | 0,19 | 0,02 |
| Ribophorin II | RPN2 | NM_002951 | 0,18 | 0,02 |
| Centromere protein A, 17kDa | CENPA | NM_001809 | 0,18 | 0,01 |
| HCF-binding transcription factor Zhangfei | ZF | NM_021212 | 0,18 | 0,00 |
| Acid phosphatase, prostate | ACPP | NM_001099 | 0,18 | 0,01 |
| Egl nine homolog 2 (C. elegans) | EGLN2 | NM_053046 | 0,18 | 0,00 |
| Chromobox homolog 3 (HP1 gamma homolog, Drosophila) | CBX3 | NM_007276 | 0,17 | 0,02 |
| ARP2 actin-related protein 2 homolog (yeast) | ACTR2 | NM_001005386 | 0,17 | 0,02 |
| Tumor necrosis factor, alpha-induced protein 2 | TNFAIP2 | NM_006291 | 0,17 | 0,00 |
| Lymphocyte cytosolic protein 1 (L-plastin) | LCP1 | NM_002298 | 0,17 | 0,02 |
| Dystonin | DST | NM_001723 | 0,17 | 0,01 |
| Non-POU domain containing, octamer-binding | NONO | NM_007363 | 0,17 | 0,01 |
| Thiosulfate sulfurtransferase (rhodanese) | TST | NM_003312 | 0,17 | 0,00 |
| Chemokine (C-X-C motif) ligand 12 (stromal cell-derived factor 1) | CXCL12 | NM_000609 | 0,17 | 0,01 |
| Enah/Vasp-like | EVL | NM_016337 | 0,17 | 0,02 |
| Adaptor-related protein complex 2, beta 1 subunit | AP2B1 | NM_001030006 | 0,16 | 0,01 |
| Caspase 1, apoptosis-related cysteine peptidase (interleukin 1, beta, convertase) | CASP1 | NM_033293 | 0,16 | 0,01 |
| Bone morphogenetic protein 1 | BMP1 | NM_001199 | 0,16 | 0,02 |
| Protease, serine, 1 (trypsin 1) | PRSS1 | NM_002769 | 0,16 | 0,00 |
| Desmuslin | DMN | NM_015286 | 0,16 | 0,02 |
| Ribosomal protein L36 | RPL36 | NM_015414 | 0,16 | 0,02 |
| Procollagen-proline, 2-oxoglutarate 4-dioxygenase (proline 4-hydroxylase), alpha polypeptide I | P4HA1 | NM_000917 | 0,16 | 0,02 |
| Eukaryotic translation elongation factor 1 beta 2 | EEF1B2 | NM_001959 | 0,16 | 0,00 |
| Stathmin-like 3 | STMN3 | NM_015894 | 0,16 | 0,01 |
| CD47 antigen (Rh-related antigen, integrin-associated signal transducer) | CD47 | NM_001025079 | 0,16 | 0,02 |
| Antigen p97 (melanoma associated) identified by monoclonal antibodies 133.2 and 96.5 | MFI2 | NM_005929 | 0,16 | 0,01 |
| Endoplasmic reticulum-golgi intermediate compartment 32 kDa protein | KIAA1181 | NM_001031711 | 0,16 | 0,01 |
| Fibroblast growth factor 13 | FGF13 | NM_033642 | 0,16 | 0,00 |
| Quaking homolog, KH domain RNA binding (mouse) | QKI | NM_206853 | 0,16 | 0,02 |
| CSE1 chromosome segregation 1-like (yeast) | CSE1L | NM_001316 | 0,15 | 0,02 |
| Nuclear receptor subfamily 5, group A, member 2 | NR5A2 | NM_003822 | 0,15 | 0,01 |
| Endoplasmic reticulum-golgi intermediate compartment 32 kDa protein | KIAA1181 | NM_020462 | 0,15 | 0,00 |
| V-erb-b2 erythroblastic leukemia viral oncogene homolog 3 (avian) | ERBB3 | NM_001982 | 0,15 | 0,01 |
| Prostaglandin-endoperoxide synthase 2 (prostaglandin G/H synthase and cyclooxygenase) | PTGS2 | NM_000963 | 0,15 | 0,01 |
| Kallikrein 12 | KLK12 | NM_019598 | 0,15 | 0,03 |
| Protective protein for beta-galactosidase (galactosialidosis) | PPGB | NM_000308 | 0,15 | 0,00 |
| Tropomyosin 4 | TPM4 | NM_003290 | 0,15 | 0,00 |
| Nuclear receptor subfamily 1, group D, member 1 | NR1D1 | NM_021724 | 0,15 | 0,00 |
| Cathepsin C | CTSC | NM_001814 | 0,15 | 0,02 |
| Gamma-aminobutyric acid (GABA) B receptor, 1 | GABBR1 | NM_021904 | 0,15 | 0,01 |
| Nuclear receptor subfamily 5, group A, member 2 | NR5A2 | NM_003822 | 0,15 | 0,01 |
| CCR4-NOT transcription complex, subunit 2 | CNOT2 | NM_014515 | 0,15 | 0,01 |
| Interleukin 1 receptor-like 1 | IL1RL1 | NM_016232 | 0,15 | 0,01 |
| Promyelocytic leukemia | PML | NM_033244 | 0,15 | 0,02 |

| D) PC3-Zn-S-NVC vs PNT1A-Zn-S-NVC |  |  |  |  |
| --- | --- | --- | --- | --- |
| UP_REGULATION |  |  |  |  |
| Name of Gene | Symbol | NCBI database | Fold_ratio | Error Standar |
| Pre-B-cell colony enhancing factor 1 | PBEF1 | NM_005746 | 5,98 | 0,45 |
| Glutaminase | GLS | NM_014905 | 5,98 | 0,26 |
| AHNAK nucleoprotein (desmoyokin) | MGC5395 | NM_024060 | 5,92 | 0,42 |
| Pyridoxal (pyridoxine, vitamin B6) kinase | PDXK | NM_003681 | 5,90 | 0,86 |
| Netrin 4 | NTN4 | NM_021229 | 5,87 | 0,90 |
| RAB6B, member RAS oncogene family | RAB6B | NM_016577 | 5,86 | 0,44 |
| Adaptor-related protein complex 2, beta 1 subunit | AP2B1 | NM_001282 | 5,83 | 1,10 |
| Amyloid beta (A4) precursor-like protein 2 | APLP2 | NM_001642 | 5,76 | 0,63 |
| Solute carrier family 2 (facilitated glucose transporter), member 3 | SLC2A3 | NM_006931 | 5,76 | 0,86 |
| Mitogen-activated protein kinase 10 | MAPK10 | NM_138980 | 5,72 | 0,94 |
| Progestin and adipoQ receptor family member III | PAQR3 | NM_177453 | 5,70 | 0,76 |
| SMC4 structural maintenance of chromosomes 4-like 1 (yeast) | SMC4L1 | NM_001002799 | 5,67 | 0,43 |
| Fibroblast growth factor 3 (murine mammary tumor virus integration site (v-int-2) oncogene homolog) | FGF3 | NM_005247 | 5,65 | 0,56 |
| Cytochrome P450, family 51, subfamily A, polypeptide 1 | CYP51A1 | NM_000786 | 5,61 | 0,67 |
| Visinin-like 1 | VSNL1 | NM_003385 | 5,46 | 0,44 |
| Fragile X mental retardation 1 | FMR1 | NM_002024 | 5,38 | 0,32 |
| Cathepsin L2 | CTSL2 | NM_001333 | 5,35 | 0,05 |
| PTK7 protein tyrosine kinase 7 | PTK7 | NM_002821 | 5,25 | 0,43 |
| Mitogen-activated protein kinase 10 | MAPK10 | NM_138980 | 5,23 | 0,03 |
| Plasminogen activator, tissue | PLAT | NM_000931 | 5,23 | 0,69 |
| Forkhead box P1 | FOXP1 | NM_001012505 | 5,11 | 0,31 |
| Peroxisomal D3,D2-enoyl-CoA isomerase | PECI | NM_006117 | 4,97 | 0,49 |
| DnaJ (Hsp40) homolog, subfamily A, member 1 | DNAJA1 | NM_001539 | 4,93 | 0,10 |
| Septin 2 | 38962 | NM_001008491 | 4,43 | 0,05 |
| Protein tyrosine phosphatase type IVA, member 2 | PTP4A2 | NM_080391 | 4,29 | 0,52 |
| Stomatin (EPB72)-like 2 | STOML2 | NM_013442 | 1,57 | 0,06 |
| MCM3 minichromosome maintenance deficient 3 (S. cerevisiae) | MCM3 | NM_002388 | 1,54 | 0,04 |
| Protease, serine, 3 (mesotrypsin) | PRSS3 | NM_002771 | 1,53 | 0,01 |
| Nuclear receptor subfamily 4, group A, member 3 | NR4A3 | NM_173199 | 1,53 | 0,02 |
| Caspase 1, apoptosis-related cysteine peptidase (interleukin 1, beta, convertase) | CASP1 | NM_033294 | 1,52 | 0,01 |
| Tripartite motif-containing 37 | TRIM37 | NM_015294 | 1,52 | 0,02 |
| Dystonin | DST | NM_015548 | 1,52 | 0,01 |
| Procollagen-proline, 2-oxoglutarate 4-dioxygenase (proline 4-hydroxylase), alpha polypeptide I | P4HA1 | NM_001017962 | 1,51 | 0,02 |
| Metadherin | LYRIC | NM_178812 | 1,51 | 0,03 |
| WNT1 inducible signaling pathway protein 1 | WISP1 | NM_080838 | 1,51 | 0,02 |
| KIAA1683 | KIAA1683 | NM_025249 | 1,51 | 0,00 |
| Transporter 1, ATP-binding cassette, sub-family B (MDR/TAP) | TAP1 | NM_000593 | 1,50 | 0,01 |
| DOWN_REGULATION |  |  |  |  |
| Name of Gene | Symbol | NCBI database | Fold_ratio | Error Standar |
| Adaptor-related protein complex 2, beta 1 subunit | AP2B1 | NM_001030006 | 0,24 | 0,01 |
| Thiosulfate sulfurtransferase (rhodanese) | TST | NM_003312 | 0,23 | 0,01 |
| Desmocollin 2 | DSC2 | NM_004949 | 0,22 | 0,00 |
| Prostaglandin-endoperoxide synthase 2 (prostaglandin G/H synthase and cyclooxygenase) | PTGS2 | NM_000963 | 0,22 | 0,01 |
| Protease, serine, 1 (trypsin 1) | PRSS1 | NM_002769 | 0,21 | 0,00 |
| Serine/threonine kinase 3 (STE20 homolog, yeast) | STK3 | NM_006281 | 0,21 | 0,01 |
| Kallikrein 12 | KLK12 | NM_019598 | 0,21 | 0,04 |
| Immunoglobulin lambda-like polypeptide 1 | IGLL1 | NM_152855 | 0,21 | 0,01 |
| Hypothetical protein FLJ21924 | FLJ21924 | NM_024774 | 0,20 | 0,00 |
| CCR4-NOT transcription complex, subunit 2 | CNOT2 | NM_014515 | 0,19 | 0,01 |
| Non-POU domain containing, octamer-binding | NONO | NM_007363 | 0,19 | 0,00 |
| Vanin 1 | VNN1 | NM_004666 | 0,18 | 0,02 |
| Egl nine homolog 2 (C. elegans) | EGLN2 | NM_053046 | 0,18 | 0,00 |
| Calnexin | CANX | NM_001024649 | 0,18 | 0,01 |
| Glycoprotein 2 (zymogen granule membrane) | GP2 | NM_001007240 | 0,17 | 0,02 |
| Centromere protein A, 17kDa | CENPA | NM_001809 | 0,17 | 0,01 |
| Dystonin | DST | NM_001723 | 0,17 | 0,02 |
| Bone morphogenetic protein 1 | BMP1 | NM_006131 | 0,17 | 0,00 |
| Lymphocyte cytosolic protein 1 (L-plastin) | LCP1 | NM_002298 | 0,17 | 0,02 |
| Bone morphogenetic protein 1 | BMP1 | NM_001199 | 0,17 | 0,01 |
| Family with sequence similarity 64, member A | FLJ10156 | NM_019013 | 0,17 | 0,04 |
| Disabled homolog 2, mitogen-responsive phosphoprotein (Drosophila) | DAB2 | NM_001343 | 0,17 | 0,02 |
| Hypothetical protein MAC30 | MAC30 | NM_014573 | 0,17 | 0,02 |
| Solute carrier family 26 (sulfate transporter), member 2 | SLC26A2 | NM_000112 | 0,17 | 0,00 |
| Desmuslin | DMN | NM_015286 | 0,17 | 0,02 |
| Tumor necrosis factor, alpha-induced protein 2 | TNFAIP2 | NM_006291 | 0,17 | 0,00 |
| Parathyroid hormone-like hormone | PTHLH | NM_198966 | 0,16 | 0,02 |
| Chromobox homolog 3 (HP1 gamma homolog, Drosophila) | CBX3 | NM_007276 | 0,16 | 0,02 |
| Chemokine (C-X-C motif) ligand 12 (stromal cell-derived factor 1) | CXCL12 | NM_000609 | 0,16 | 0,01 |
| Endoplasmic reticulum-golgi intermediate compartment 32 kDa protein | KIAA1181 | NM_001031711 | 0,16 | 0,01 |
| Stathmin-like 3 | STMN3 | NM_015894 | 0,16 | 0,00 |
| DAB2 interacting protein | DAB2IP | NM_032552 | 0,16 | 0,01 |
| Ribophorin II | RPN2 | NM_002951 | 0,16 | 0,01 |
| Fibroblast growth factor 13 | FGF13 | NM_033642 | 0,16 | 0,00 |
| Enah/Vasp-like | EVL | NM_016337 | 0,16 | 0,01 |
| CD47 antigen (Rh-related antigen, integrin-associated signal transducer) | CD47 | NM_001025079 | 0,16 | 0,02 |
| CSE1 chromosome segregation 1-like (yeast) | CSE1L | NM_001316 | 0,16 | 0,02 |
| Tropomyosin 4 | TPM4 | NM_003290 | 0,16 | 0,00 |
| Potassium large conductance calcium-activated channel, subfamily M, alpha member 1 | KCNMA1 | NM_002247 | 0,16 | 0,01 |
| Nuclear receptor subfamily 5, group A, member 2 | NR5A2 | NM_003822 | 0,15 | 0,01 |
| Antigen p97 (melanoma associated) identified by monoclonal antibodies 133.2 and 96.5 | MFI2 | NM_005929 | 0,15 | 0,00 |
| High-mobility group box 3 | HMGB3 | NM_005342 | 0,15 | 0,02 |
| Nuclear receptor subfamily 5, group A, member 2 | NR5A2 | NM_003822 | 0,15 | 0,00 |
| Gamma-aminobutyric acid (GABA) B receptor, 1 | GABBR1 | NM_021904 | 0,15 | 0,00 |
| Ribosomal protein L36 | RPL36 | NM_015414 | 0,15 | 0,02 |
| Protective protein for beta-galactosidase (galactosialidosis) | PPGB | NM_000308 | 0,15 | 0,00 |
| Collagen, type V, alpha 2 | COL5A2 | NM_000393 | 0,15 | 0,00 |
| Endoplasmic reticulum-golgi intermediate compartment 32 kDa protein | KIAA1181 | NM_020462 | 0,15 | 0,00 |
| Cathepsin C | CTSC | NM_001814 | 0,15 | 0,02 |

Supplementary Table C. The lists of biological processes of up and/or down regulation genes in PC3 and PNT1A cells lines after treatment Zn-S-NVC complex by Gene Ontology (GO) annotations. A) PC3 vs PNT1A. B) PNT1A-Zn-S-NVC vs PNT1A. C) PC3-Zn-S-NVC vs PC3. D) PC3-Zn-S-NVC vs PNT1A-Zn-S-NVC.

| A) PC3 vs PNT1A. |  |  |  |
| --- | --- | --- | --- |
| UP_REGULATION | Annotation_Process_GO |  |  |
| GO_id | Term | Number Of Genes | p-value |
| GO:0065007 | biological regulation | 34 | 1,32E-02 |
| GO:0050896 | response to stimulus | 33 | 6,90E-05 |
| GO:0051716 | cellular response to stimulus | 32 | 5,59E-06 |
| GO:0042221 | response to chemical | 30 | 4,63E-09 |
| GO:0044700 | single organism signaling | 29 | 1,04E-05 |
| GO:0007165 | signal transduction | 28 | 6,00E-06 |
| GO:0051171 | regulation of nitrogen compound metabolic process | 27 | 4,96E-07 |
| GO:0006950 | response to stress | 25 | 6,77E-07 |
| GO:0031326 | regulation of cellular biosynthetic process | 24 | 2,68E-05 |
| GO:0010556 | regulation of macromolecule biosynthetic process | 23 | 4,04E-05 |
| GO:0051252 | regulation of RNA metabolic process | 22 | 3,34E-05 |
| GO:0010033 | response to organic substance | 21 | 9,35E-07 |
| GO:0006355 | regulation of transcription, DNA-templated | 20 | 2,17E-04 |
| GO:0007166 | cell surface receptor signaling pathway | 19 | 2,41E-04 |
| GO:0009893 | positive regulation of metabolic process | 18 | 1,50E-03 |
| GO:0010604 | positive regulation of macromolecule metabolic process | 17 | 2,76E-04 |
| GO:0070887 | cellular response to chemical stimulus | 16 | 2,93E-04 |
| GO:0022403 | cell cycle phase | 15 | 1,74E-14 |
| GO:0071310 | cellular response to organic substance | 14 | 3,80E-04 |
| GO:0007264 | small GTPase mediated signal transduction | 13 | 1,12E-08 |
| GO:0007167 | enzyme linked receptor protein signaling pathway | 11 | 4,86E-05 |
| GO:0000084 | mitotic S phase | 10 | 2,87E-12 |
| GO:0000075 | cell cycle checkpoint | 9 | 7,24E-08 |
| GO:0060548 | negative regulation of cell death | 8 | 3,94E-03 |
| GO:0051253 | negative regulation of RNA metabolic process | 7 | 2,88E-02 |
| GO:0040008 | regulation of growth | 6 | 8,62E-03 |
| GO:0007067 | mitotic nuclear division | 5 | 3,21E-03 |
| GO:0050000 | chromosome localization | 4 | 1,59E-05 |
| DOWN_REGULATION | Annotation_Process_GO |  |  |
| GO_id | Term | Number Of Genes | p-value |
| GO:0044237 | cellular metabolic process | 24 | 7,88E-06 |
| GO:0007165 | signal transduction | 23 | 5,47E-09 |
| GO:0009059 | macromolecule biosynthetic process | 22 | 9,50E-10 |
| GO:0046483 | heterocycle metabolic process | 21 | 1,85E-07 |
| GO:0090304 | nucleic acid metabolic process | 20 | 1,02E-07 |
| GO:0016070 | RNA metabolic process | 19 | 1,36E-07 |
| GO:0022403 | cell cycle phase | 18 | 4,10E-24 |
| GO:0010467 | gene expression | 17 | 9,47E-06 |
| GO:0033554 | cellular response to stress | 16 | 1,55E-10 |
| GO:0000278 | mitotic cell cycle | 15 | 6,60E-13 |
| GO:0007049 | cell cycle | 14 | 5,62E-09 |
| GO:0022402 | cell cycle process | 13 | 2,19E-09 |
| GO:1903047 | mitotic cell cycle process | 12 | 7,44E-10 |
| GO:0009889 | regulation of biosynthetic process | 12 | 8,71E-03 |
| GO:0018193 | peptidyl-amino acid modification | 11 | 1,85E-08 |
| GO:0051329 | mitotic interphase | 10 | 1,94E-14 |
| GO:0000080 | mitotic G1 phase | 9 | 1,41E-18 |
| GO:0000088 | mitotic prophase | 8 | 1,84E-13 |
| GO:0007077 | mitotic nuclear envelope disassembly | 7 | 8,94E-13 |
| GO:0000082 | G1/S transition of mitotic cell cycle | 6 | 5,66E-07 |
| GO:0007179 | transforming growth factor beta receptor signaling pathway | 5 | 2,71E-06 |
| GO:0043065 | positive regulation of apoptotic process | 2 | 2,41E-01 |
| GO:0043068 | positive regulation of programmed cell death | 2 | 2,45E-01 |

| B) PNT1A-Zn-S-NVC vs PNT1A. | |  |  |
| --- | --- | --- | --- |
| UP_REGULATION | Annotation_Process_GO |  |  |
| GO_id | Term | Number Of Genes | p-value |
| GO:0050896 | response to stimulus | 38 | 1,18E-10 |
| GO:0051716 | cellular response to stimulus | 35 | 1,60E-10 |
| GO:0065007 | biological regulation | 33 | 5,41E-04 |
| GO:0050794 | regulation of cellular process | 26 | 4,15E-02 |
| GO:0016043 | cellular component organization | 25 | 1,14E-05 |
| GO:0044700 | single organism signaling | 24 | 6,76E-05 |
| GO:0048523 | negative regulation of cellular process | 23 | 2,80E-06 |
| GO:0044238 | primary metabolic process | 22 | 5,81E-02 |
| GO:0022403 | cell cycle phase | 21 | 3,73E-25 |
| GO:0065008 | regulation of biological quality | 20 | 5,55E-06 |
| GO:1902589 | single-organism organelle organization | 19 | 1,46E-08 |
| GO:0000278 | mitotic cell cycle | 18 | 2,54E-13 |
| GO:0022402 | cell cycle process | 17 | 7,56E-11 |
| GO:0042221 | response to chemical | 16 | 6,21E-03 |
| GO:0035556 | intracellular signal transduction | 15 | 5,27E-06 |
| GO:0010033 | response to organic substance | 14 | 7,98E-04 |
| GO:0000075 | cell cycle checkpoint | 13 | 5,12E-14 |
| GO:0033554 | cellular response to stress | 13 | 2,30E-05 |
| GO:0000087 | mitotic M phase | 12 | 4,64E-13 |
| GO:0070887 | cellular response to chemical stimulus | 12 | 4,17E-03 |
| GO:0045786 | negative regulation of cell cycle | 11 | 1,05E-08 |
| GO:0071310 | cellular response to organic substance | 10 | 8,01E-03 |
| GO:0000084 | mitotic S phase | 9 | 1,78E-11 |
| GO:0032201 | telomere maintenance via semi-conservative replication | 8 | 1,14E-15 |
| GO:0051782 | negative regulation of cell division | 7 | 5,25E-10 |
| GO:0070987 | error-free translesion synthesis | 6 | 5,16E-12 |
| GO:0042276 | error-prone translesion synthesis | 5 | 1,32E-09 |
| GO:0000070 | mitotic sister chromatid segregation | 4 | 3,16E-05 |
| DOWN_REGULATION | Annotation_Process_GO |  |  |
| GO_id | Term | Number Of Genes | p-value |
| GO:0032501 | multicellular organismal process | 18 | 6,07E-04 |
| GO:1901698 | response to nitrogen compound | 17 | 1,50E-15 |
| GO:0010243 | response to organonitrogen compound | 16 | 8,42E-15 |
| GO:0038095 | Fc-epsilon receptor signaling pathway | 15 | 2,28E-22 |
| GO:0051240 | positive regulation of multicellular organismal process | 14 | 1,48E-09 |
| GO:0032870 | cellular response to hormone stimulus | 13 | 1,32E-12 |
| GO:0051094 | positive regulation of developmental process | 12 | 1,94E-08 |
| GO:0010647 | positive regulation of cell communication | 11 | 6,65E-06 |
| GO:0048017 | inositol lipid-mediated signaling | 10 | 1,32E-14 |
| GO:0010941 | regulation of cell death | 9 | 2,64E-04 |
| GO:0065007 | biological regulation | 8 | 9,83E-01 |
| GO:0051781 | positive regulation of cell division | 7 | 1,22E-09 |
| GO:0070374 | positive regulation of ERK1 and ERK2 cascade | 6 | 4,95E-08 |
| GO:0001759 | organ induction | 5 | 1,44E-10 |
| GO:0060484 | positive regulation of lipid metabolic process | 4 | 2,81E-05 |

| C) PC3-Zn-S-NVC vs PC3. | |  |  |
| --- | --- | --- | --- |
| UP_REGULATION | Annotation_Process_GO |  |  |
| GO_id | Term | Number Of Genes | p-value |
| GO:0044763 | single-organism cellular process | 37 | 1,44E-03 |
| GO:0044699 | single-organism process | 36 | 6,31E-03 |
| GO:0006807 | nitrogen compound metabolic process | 33 | 1,04E-07 |
| GO:0016043 | cellular component organization | 32 | 7,03E-08 |
| GO:0034641 | cellular nitrogen compound metabolic process | 30 | 1,09E-06 |
| GO:0046483 | heterocycle metabolic process | 29 | 8,89E-07 |
| GO:0006139 | nucleobase-containing compound metabolic process | 28 | 1,46E-06 |
| GO:0048519 | negative regulation of biological process | 27 | 2,16E-06 |
| GO:0032774 | RNA biosynthetic process | 26 | 1,04E-08 |
| GO:0051179 | localization | 25 | 2,21E-05 |
| GO:0051704 | multi-organism process | 24 | 1,10E-08 |
| GO:1901575 | organic substance catabolic process | 23 | 1,10E-12 |
| GO:0071822 | protein complex subunit organization | 22 | 1,82E-11 |
| GO:0009057 | macromolecule catabolic process | 21 | 5,61E-15 |
| GO:0044238 | primary metabolic process | 21 | 3,40E-01 |
| GO:0022411 | cellular component disassembly | 20 | 2,87E-17 |
| GO:0031324 | negative regulation of cellular metabolic process | 20 | 2,79E-07 |
| GO:0033365 | protein localization to organelle | 19 | 2,56E-16 |
| GO:0008152 | metabolic process | 19 | 7,34E-01 |
| GO:0006413 | translational initiation | 18 | 4,18E-21 |
| GO:0006614 | SRP-dependent cotranslational protein targeting to membrane | 17 | 2,76E-25 |
| GO:0002376 | immune system process | 16 | 1,09E-04 |
| GO:0006950 | response to stress | 16 | 1,17E-02 |
| GO:0044267 | cellular protein metabolic process | 15 | 4,75E-03 |
| GO:0010605 | negative regulation of macromolecule metabolic process | 14 | 6,82E-04 |
| GO:0048585 | negative regulation of response to stimulus | 13 | 4,17E-05 |
| GO:0045934 | negative regulation of nucleobase-containing compound metabolic process | 12 | 7,71E-05 |
| GO:0010468 | regulation of gene expression | 11 | 4,49E-01 |
| GO:0051253 | negative regulation of RNA metabolic process | 10 | 6,25E-04 |
| GO:2000113 | negative regulation of cellular macromolecule biosynthetic process | 9 | 3,14E-03 |
| GO:0042981 | regulation of apoptotic process | 8 | 3,66E-02 |
| GO:0000122 | negative regulation of transcription from RNA polymerase II promoter | 7 | 3,36E-03 |
| GO:0034470 | ncRNA processing | 6 | 9,88E-05 |
| GO:0012501 | programmed cell death | 5 | 1,96E-01 |
| GO:0045936 | negative regulation of phosphate metabolic process | 4 | 4,49E-02 |
| GO:0043065 | positive regulation of apoptotic process | 3 | 2,28E-01 |
| DOWN_REGULATION | Annotation_Process_GO |  |  |
| GO_id | Term | Number Of Genes | p-value |
| GO:0044702 | cellular process | 34 | 2,42E-01 |
| GO:0019882 | single-organism cellular process | 32 | 1,12E-01 |
| GO:1902578 | nitrogen compound metabolic process | 29 | 8,60E-05 |
| GO:0001503 | cellular component organization or biogenesis | 28 | 9,89E-05 |
| GO:0032990 | single-organism developmental process | 27 | 1,77E-04 |
| GO:0010648 | localization | 26 | 3,13E-05 |
| GO:0006259 | response to stimulus | 25 | 7,46E-02 |
| GO:0022008 | cellular aromatic compound metabolic process | 24 | 1,03E-03 |
| GO:0001666 | multicellular organismal development | 23 | 1,35E-03 |
| GO:0017148 | positive regulation of biological process | 23 | 5,48E-03 |
| GO:0050729 | gene expression | 22 | 5,18E-04 |
| GO:1900024 | cellular component biogenesis | 21 | 1,09E-07 |
| GO:0045927 | cell differentiation | 20 | 5,79E-04 |
| GO:0044248 | cellular catabolic process | 19 | 7,19E-09 |
| GO:0016071 | mRNA metabolic process | 18 | 1,14E-13 |
| GO:0044270 | cellular nitrogen compound catabolic process | 17 | 3,15E-15 |
| GO:0000956 | nuclear-transcribed mRNA catabolic process | 16 | 5,64E-19 |
| GO:0002429 | positive regulation of response to stimulus | 15 | 2,28E-04 |
| GO:0043065 | positive regulation of metabolic process | 14 | 6,50E-02 |
| GO:0033143 | protein transport | 13 | 6,11E-05 |
| GO:0045785 | defense response | 12 | 7,56E-04 |
| GO:0042254 | ribosome biogenesis | 11 | 1,52E-11 |
| GO:0051603 | positive regulation of cellular metabolic process | 10 | 2,20E-01 |
| GO:0090503 | RNA phosphodiester bond hydrolysis, exonucleolytic | 9 | 3,39E-18 |
| GO:0030307 | regulation of cell growth | 8 | 1,31E-05 |
| GO:0010811 | positive regulation of cellular component organization | 7 | 1,91E-02 |
| GO:0051094 | anatomical structure formation involved in morphogenesis | 6 | 5,60E-02 |
| GO:0010720 | positive regulation of developmental process | 6 | 8,23E-02 |
| GO:0009719 | positive regulation of cell differentiation | 5 | 7,42E-02 |
| GO:0045862 | negative regulation of apoptotic signaling pathway | 4 | 4,18E-03 |

| D) PC3-Zn-S-NVC vs PNT1A-Zn-S-NVC. | |  |  |
| --- | --- | --- | --- |
| UP_REGULATION | Annotation_Process_GO |  |  |
| GO_id | Term | Number Of Genes | p-value |
| GO:0044763 | single-organism cellular process | 27 | 6,01E-02 |
| GO:0016043 | cellular component organization | 25 | 1,14E-05 |
| GO:0044699 | single-organism process | 24 | 3,01E-01 |
| GO:0031323 | regulation of cellular metabolic process | 18 | 3,92E-02 |
| GO:0022403 | cell cycle phase | 17 | 9,35E-19 |
| GO:0000278 | mitotic cell cycle | 16 | 4,13E-11 |
| GO:1903047 | mitotic cell cycle process | 15 | 8,92E-11 |
| GO:0046483 | heterocycle metabolic process | 15 | 6,50E-02 |
| GO:0042221 | response to chemical | 14 | 3,21E-02 |
| GO:0051128 | regulation of cellular component organization | 13 | 7,47E-04 |
| GO:0065009 | regulation of molecular function | 12 | 9,95E-03 |
| GO:0031324 | negative regulation of cellular metabolic process | 11 | 6,37E-03 |
| GO:0071310 | cellular response to organic substance | 10 | 8,01E-03 |
| GO:0019220 | regulation of phosphate metabolic process | 9 | 7,43E-03 |
| GO:0010941 | regulation of cell death | 8 | 1,91E-02 |
| GO:0043067 | regulation of programmed cell death | 7 | 4,32E-02 |
| GO:0006261 | DNA-dependent DNA replication | 6 | 4,23E-08 |
| GO:0002764 | immune response-regulating signaling pathway | 6 | 1,46E-03 |
| GO:0006260 | DNA replication | 5 | 1,01E-04 |
| GO:0010628 | positive regulation of gene expression | 4 | 4,69E-01 |
| DOWN_REGULATION | Annotation_Process_GO |  |  |
| GO_id | Term | Number Of Genes | p-value |
| GO:0044763 | single-organism cellular process | 42 | 3,46E-05 |
| GO:0044699 | single-organism process | 39 | 1,35E-03 |
| GO:0016043 | cellular component organization | 33 | 4,34E-08 |
| GO:0051179 | localization | 32 | 3,49E-09 |
| GO:0006807 | nitrogen compound metabolic process | 31 | 3,28E-06 |
| GO:0033036 | macromolecule localization | 30 | 2,60E-15 |
| GO:0006810 | transport | 29 | 1,25E-09 |
| GO:0051641 | cellular localization | 28 | 6,78E-13 |
| GO:0034613 | cellular protein localization | 27 | 1,30E-18 |
| GO:0015031 | protein transport | 26 | 1,29E-16 |
| GO:0009987 | cellular process | 25 | 8,82E-01 |
| GO:0022411 | cellular component disassembly | 24 | 2,87E-22 |
| GO:0090304 | nucleic acid metabolic process | 23 | 1,65E-04 |
| GO:0072657 | protein localization to membrane | 22 | 1,61E-23 |
| GO:0006614 | SRP-dependent cotranslational protein targeting to membrane | 21 | 5,42E-33 |
| GO:0043624 | cellular protein complex disassembly | 20 | 4,50E-25 |
| GO:0006415 | translational termination | 19 | 1,44E-24 |
| GO:0050896 | response to stimulus | 18 | 5,74E-01 |
| GO:0007275 | multicellular organismal development | 17 | 6,69E-02 |
| GO:0050789 | regulation of biological process | 16 | 9,88E-01 |
| GO:0048518 | positive regulation of biological process | 15 | 3,16E-01 |
| GO:0030154 | cell differentiation | 14 | 5,51E-02 |
| GO:0048522 | positive regulation of cellular process | 13 | 3,50E-01 |
| GO:0080090 | regulation of primary metabolic process | 12 | 7,42E-01 |
| GO:0044238 | primary metabolic process | 11 | 9,95E-01 |
| GO:0006928 | movement of cell or subcellular component | 10 | 4,40E-03 |
| GO:0080134 | regulation of response to stress | 9 | 6,69E-03 |
| GO:0031326 | regulation of cellular biosynthetic process | 8 | 8,35E-01 |
| GO:0031325 | positive regulation of cellular metabolic process | 7 | 5,95E-01 |
| GO:0040008 | regulation of growth | 6 | 9,26E-03 |
| GO:0006412 | translation | 5 | 1,71E-03 |
| GO:0042273 | ribosomal large subunit biogenesis | 4 | 3,11E-07 |

Supplementary Table D. The lists of pathways of up and/or down regulation genes in PC3 and PNT1A cells lines after treatment Zn-S-NVC complex by KEGG 10 software. A) PC3 vs PNT1A. B) PNT1A-Zn-S-NVC vs PNT1A. C) PC3-Zn-S-NVC vs PC3. D) PC3-Zn-S-NVC vs PNT1A-Zn-S-NVC.

| A) PC3 vs PNT1A. |  |  |  |
| --- | --- | --- | --- |
| UP_REGULATION | Annotation_KEGG_Pathway |  |  |
| Id | Term | Number Of Genes | p-value |
| 5200 | Pathways in cancer | 8 | 8,58E-06 |
| 3030 | DNA replication | 7 | 1,64E-11 |
| 3430 | Mismatch repair | 6 | 8,64E-11 |
| 5206 | MicroRNAs in cancer | 5 | 9,87E-05 |
| 5215 | Prostate cancer | 4 | 1,64E-04 |
| 4110 | Cell cycle | 4 | 6,32E-04 |
| 5202 | Transcriptional misregulation in cancer | 3 | 1,60E-02 |
| 4115 | p53 signaling pathway | 2 | 1,93E-02 |
| 5205 | Proteoglycans in cancer | 2 | 1,56E-01 |
| 480 | Glutathione metabolism | 1 | 1,00E+00 |
| 5204 | Chemical carcinogenesis | 1 | 1,00E+00 |
| 2010 | ABC transporters | 1 | 1,00E+00 |
| 5034 | Alcoholism | 1 | 1,00E+00 |
| 564 | Glycerophospholipid metabolism | 1 | 1,00E+00 |
| 4650 | Natural killer cell mediated cytotoxicity | 1 | 1,00E+00 |
| 982 | Drug metabolism - cytochrome P450 | 1 | 1,00E+00 |
| DOWN_REGULATION | Annotation_KEGG_Pathway |  |  |
| Id | Term | Number Of Genes | p-value |
| 5205 | Proteoglycans in cancer | 2 | 1,38E-02 |
| 5200 | Pathways in cancer | 1 | 1,00E+00 |
| 4110 | Cell cycle | 1 | 1,00E+00 |
| 4974 | Protein digestion and absorption | 1 | 1,00E+00 |
| 3013 | RNA transport | 1 | 1,00E+00 |
| 5206 | MicroRNAs in cancer | 1 | 1,00E+00 |

| B) PNT1A-Zn-S-NVC vs PNT1A. | |  |  |
| --- | --- | --- | --- |
| UP_REGULATION | Annotation_KEGG_Pathway |  |  |
| Id | Term | Number Of Genes | p-value |
| 3030 | DNA replication | 6 | 1,24E-10 |
| 3430 | Mismatch repair | 5 | 1,34E-09 |
| 3420 | Nucleotide excision repair | 5 | 5,25E-08 |
| 4110 | Cell cycle | 5 | 6,74E-06 |
| 5200 | Pathways in cancer | 4 | 5,29E-03 |
| 1100 | Metabolic pathways | 3 | 4,74E-01 |
| 5202 | Transcriptional misregulation in cancer | 2 | 5,05E-02 |
| 5203 | Viral carcinogenesis | 2 | 6,02E-02 |
| 1200 | Carbon metabolism | 1 | 1,00E+00 |
| 10 | Glycolysis / Gluconeogenesis | 1 | 1,00E+00 |
| 480 | Glutathione metabolism | 1 | 1,00E+00 |
| 2010 | ABC transporters | 1 | 1,00E+00 |
| 980 | Metabolism of xenobiotics by cytochrome P450 | 1 | 1,00E+00 |
| DOWN_REGULATION | Annotation_KEGG_Pathway |  |  |
| Id | Term | Number Of Genes | p-value |
| 4014 | Ras signaling pathway | 2 | 1,24E-02 |
| 5200 | Pathways in cancer | 2 | 2,53E-02 |
| 4620 | Toll-like receptor signaling pathway | 1 | 1,00E+00 |
| 4668 | TNF signaling pathway | 1 | 1,00E+00 |
| 5164 | Influenza A | 1 | 1,00E+00 |
| 4012 | ErbB signaling pathway | 1 | 1,00E+00 |
| 5205 | Proteoglycans in cancer | 1 | 1,00E+00 |
| 1100 | Metabolic pathways | 1 | 1,00E+00 |

| C) PC3-Zn-S-NVC vs PC3. |  |  |  |
| --- | --- | --- | --- |
| UP_REGULATION | Annotation_KEGG_Pathway |  |  |
| Id | Term | Number Of Genes | p-value |
| 3010 | Ribosome | 7 | 5,03E-08 |
| 5206 | MicroRNAs in cancer | 4 | 5,92E-04 |
| 1100 | Metabolic pathways | 4 | 3,81E-01 |
| 5202 | Transcriptional misregulation in cancer | 3 | 9,78E-03 |
| 5200 | Pathways in cancer | 2 | 2,13E-01 |
| 4978 | Mineral absorption | 1 | 1,00E+00 |
| 5204 | Chemical carcinogenesis | 1 | 1,00E+00 |
| 983 | Drug metabolism - other enzymes | 1 | 1,00E+00 |
| 982 | Drug metabolism - cytochrome P450 | 1 | 1,00E+00 |
| 980 | Metabolism of xenobiotics by cytochrome P450 | 1 | 1,00E+00 |
| 3050 | Proteasome | 1 | 1,00E+00 |
| 500 | Starch and sucrose metabolism | 1 | 1,00E+00 |
| DOWN_REGULATION | Annotation_KEGG_Pathway |  |  |
| Id | Term | Number Of Genes | p-value |
| 3018 | RNA degradation | 7 | 1,49E-09 |
| 5200 | Pathways in cancer | 4 | 1,44E-02 |
| 1100 | Metabolic pathways | 4 | 4,45E-01 |
| 4810 | Regulation of actin cytoskeleton | 3 | 2,32E-02 |
| 3010 | Ribosome | 2 | 5,43E-02 |
| 5206 | MicroRNAs in cancer | 1 | 1,00E+00 |
| 4122 | Sulfur relay system | 1 | 1,00E+00 |

| D) PC3-Zn-S-NVC vs PNT1A-Zn-S-NVC. |  |  |  |
| --- | --- | --- | --- |
| UP_REGULATION | Annotation_KEGG_Pathway |  |  |
| Id | Term | Number Of Genes | p-value |
| 4110 | Cell cycle | 12 | 3,72E-16 |
| 3030 | DNA replication | 6 | 4,36E-10 |
| 4010 | MAPK signaling pathway | 5 | 5,64E-04 |
| 5200 | Pathways in cancer | 5 | 1,67E-03 |
| 1100 | Metabolic pathways | 4 | 3,81E-01 |
| 4810 | Regulation of actin cytoskeleton | 3 | 1,85E-02 |
| 4151 | PI3K-Akt signaling pathway | 3 | 6,17E-02 |
| 5206 | MicroRNAs in cancer | 2 | 5,66E-02 |
| 5202 | Transcriptional misregulation in cancer | 2 | 7,20E-02 |
| 5205 | Proteoglycans in cancer | 2 | 1,16E-01 |
| 5215 | Prostate cancer | 1 | 1,00E+00 |
| 4750 | Inflammatory mediator regulation of TRP channels | 1 | 1,00E+00 |
| DOWN_REGULATION | Annotation_KEGG_Pathway |  |  |
| Id | Term | Number Of Genes | p-value |
| 3010 | Ribosome | 9 | 7,72E-11 |
| 1100 | Metabolic pathways | 4 | 4,07E-01 |
| 5200 | Pathways in cancer | 3 | 5,98E-02 |
| 510 | N-Glycan biosynthesis | 2 | 7,93E-03 |
| 3018 | RNA degradation | 2 | 1,68E-02 |
| 5204 | Chemical carcinogenesis | 1 | 1,00E+00 |
| 5206 | MicroRNAs in cancer | 1 | 1,00E+00 |
| 5205 | Proteoglycans in cancer | 1 | 1,00E+00 |

Supplementary **Note**. **Electrochemical characterization of the Zn-S-NVC complex.**

Electrochemical measurements were performed with the AUTOLAB Analyzer (EcoChemie, Netherlands) connected to VA-Stand 663 (Metrohm, Switzerland), using a standard cell with three electrodes. The working electrode was a hanging mercury drop electrode (HMDE) with a drop area of 0.4 mm^2^. The reference electrode was the Ag/AgCl/3M KCl electrode, and the auxiliary electrode was the graphite electrode (GE). The analyzed samples were deoxygenated prior to measurements by purging with argon (99.999%) and then saturated with water for 120 s. All experiments were performed at 25 °C. For smoothing and baseline correction, GPES 4.4 software supplied by EcoChemie was employed.

The interaction of NVC and Zn-S was studied by cyclic voltammetry in the presence of Britton Robinson buffer (pH 7). The parameters of the measurement by cyclic voltammetry were as follows: initial potential of −1.7 V, first vertex potential of 0.2 V, second vertex potential of −1.7 V, deposition time of 60 s, deposition potential of −1.7 V, voltage step of 5 mV, and sweep rate of 1 V·s^-1^. The volume of the injected sample was 20 µL, and the volume of the measurement cell was 2 mL (20 μL of sample+1980 µL of buffer).

Furthermore, the interaction of NVC with Zn-S was confirmed and studied using electrochemical methods. To achieve this goal, cyclic voltammetry, the most complex method, was used. The obtained voltammograms for the prepared complex and its individual components are presented in Fig. 3c. The results revealed three visible basic peaks as follows: O1 as an oxidation peak of zinc and R1 and R2 as reduction peaks of zinc in various forms/environments. Cyclic voltammograms related to the pure NVC showed only a reduction peak R1 at a potential of -1.25 V, which can be related to the reduction of free Zn^2+^ ions in the NVC solution. By measuring Zn-S, two peaks were observed: an oxidation peak O1 at potential -0.85 V, which was related to the oxidation of zinc bound to the structure of Zn-S; and a peak at approximately -1.5 V related to the reduction of zinc ions present in Zn-S.

Interactions between Zn-S and NVC were electrochemically studied using two basic techniques. The first was similar to previous determinations of individual parts. Here, the obtained voltammogram contained three peaks. The first was an oxidation peak O1, which was distinctly shifted to a more positive potential, to -0.65 V, in relation to the peak of -0.85 V for pure Zn-S. This shift indicated easier oxidation of zinc in the Zn-S-NVC complex and easier electron transfer through oxidation processes due to the structure of the created complex. However, both reduction peaks, R1 and R2, were observed. Each peak was related to the reduction of zinc ions in individual forms, NVC and Zn-S, respectively. Here, it is interesting that only the potential of the reduction peak R1 was slightly influenced by the complex formation, in relation to the individual parts. Peak R1, which is the reduction peak of zinc present in peptide solution, was shifted to more positive values (to -1.16 V) compared with pure NVC (-1.25 V). This phenomenon indicates that the presence of Zn-S with NVC slightly influenced the behavior of zinc ions and confirms the basic suggestion that these zinc ions are not closely associated with NVC molecules. A different situation was observed during the second reduction process (peak R2), which is related to the zinc located in the structure of Zn-S, in which the peak potential after the interaction with NVC remained unchanged. Here, no potential shift was observed, and thus, no influence of the zinc ion incorporated in Zn-S structure through the NVC was predicted. This result suggests that individual parts of the complex (Zn-S + NVC) are not bounded through the zinc site of Zn-S. The various intensities of peaks R1 and R2 related to the individual variants of samples were caused by the different adsorption properties of NVC and Zn-S on an electrode surface. The proteins are adsorbed better on the mercury electrode than on simple molecules [[1](#_ENREF_1),[2](#_ENREF_2),[3](#_ENREF_3)][,](#page23) as observed for Zn-S.

The second method applied for the interaction study between Zn-S and NVC was based on the utilization of the double adsorptive transfer technique (DAdT), which ensured the determination of analytes on the electrode modified in previous step by another substance. The double adsorptive transfer technique (DAdT) is based on the strong successive adsorption of both analytes on the electrode surface in an open electrode circuit (60 s). The excess analytes are rinsed from the surface of the working electrode in the buffer. The adsorbed analyte is subsequently detected in the presence of the supporting electrolyte. Specifically, the electrode surface was first modified with NVC because, in general, proteins/peptides have a higher affinity for mercury electrodes [[1](#_ENREF_1),[2](#_ENREF_2),[3](#_ENREF_3)][.](#page23) In the second step, Zn-S was adsorbed on an electrode that had been previously modified with NVC. Therefore, the interaction between NVC and Zn-S was the only reason for the adsorption of Zn-S on the modified electrode surface. Additionally, because all three of the abovementioned signals (O1, R1, R2) were observed on the obtained voltammogram (Fig. 3c), and the R2 peak, which is linked to the zinc located in Zn-S, was the most intense, it can be concluded that Zn-S interacted with NVC. The individual potentials of peaks detected utilizing DAdT were the same as in the absence of DAdT. Thus, this second interaction study method confirms the suggestion that Zn-S and NVC create a complex.

References

1. Permentier HP, Bruins AP, Bischoff R (2008) Electrochemistry-mass Spectrometry in drug metabolism and protein research. Mini-Reviews in Medicinal Chemistry 8: 46-56.

2. Palecek E, Tkac J, Bartosik M, Bertok T, Ostatna V, et al. (2015) Electrochemistry of Nonconjugated Proteins and Glycoproteins. Toward Sensors for Biomedicine and Glycomics. Chemical Reviews 115: 2045-2108.

3. Melin F, Hellwig P (2013) Recent advances in the electrochemistry and spectroelectrochemistry of membrane proteins. Biological Chemistry 394: 593-609.
