# Supplementary figures and images for: Correction: The Zinc-Schiff Base-Novicidin Complex as a Potential Prostate Cancer Therapy
Source: PLoS One. 2022 Jun 24;17(6):e0270734. doi: 10.1371/journal.pone.0270734 (PMC9231690; doi:10.1371/journal.pone.0270734)

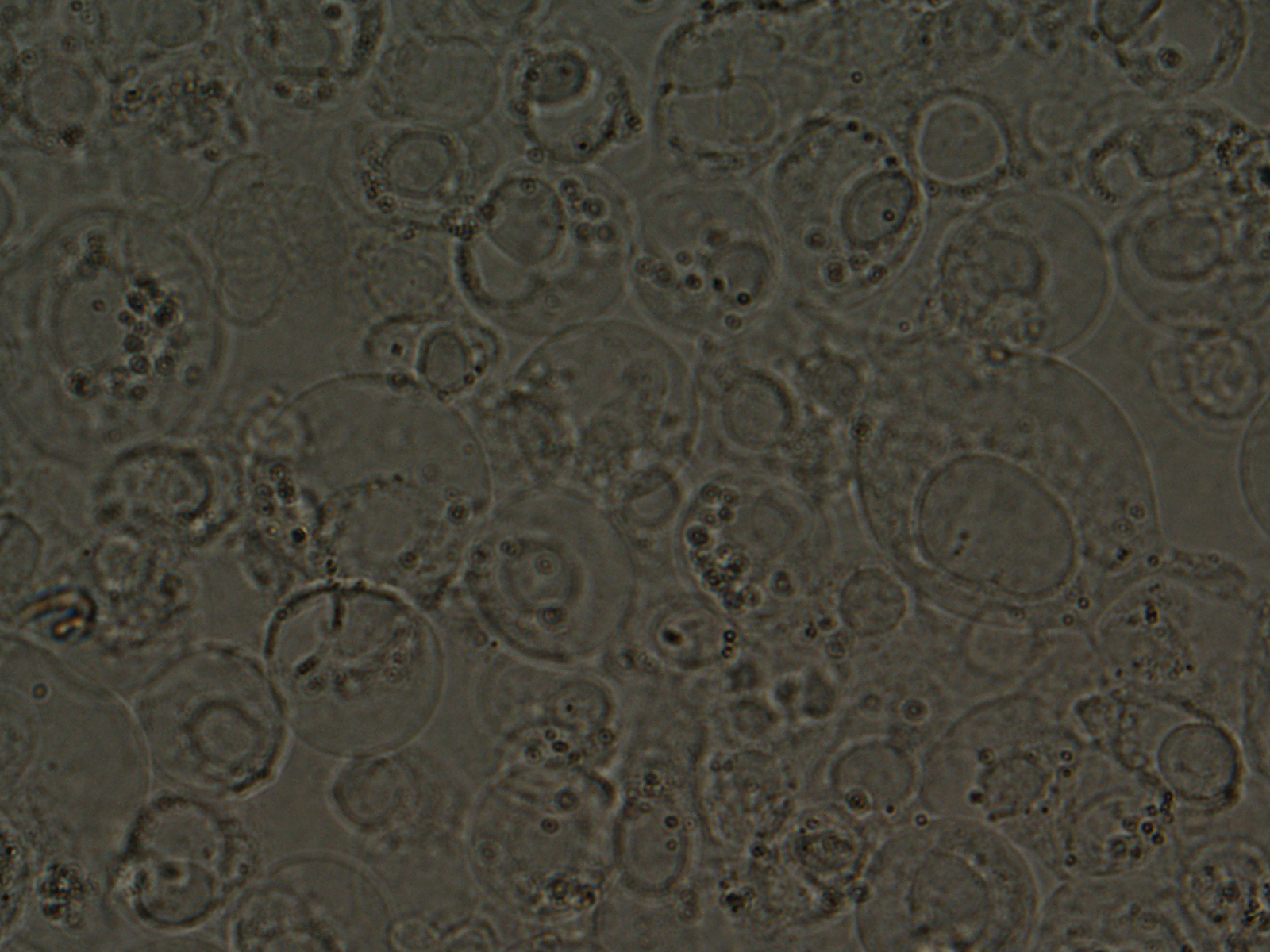

Supplement: S1 File — (ZIP) [file pone.0270734.s001.zip › S1 File/Figure 4A_PC3 cells exposed to Zn-S-NVC (conjugated to fluorescent dye) at 0 min/Brightfield_Image.tif]

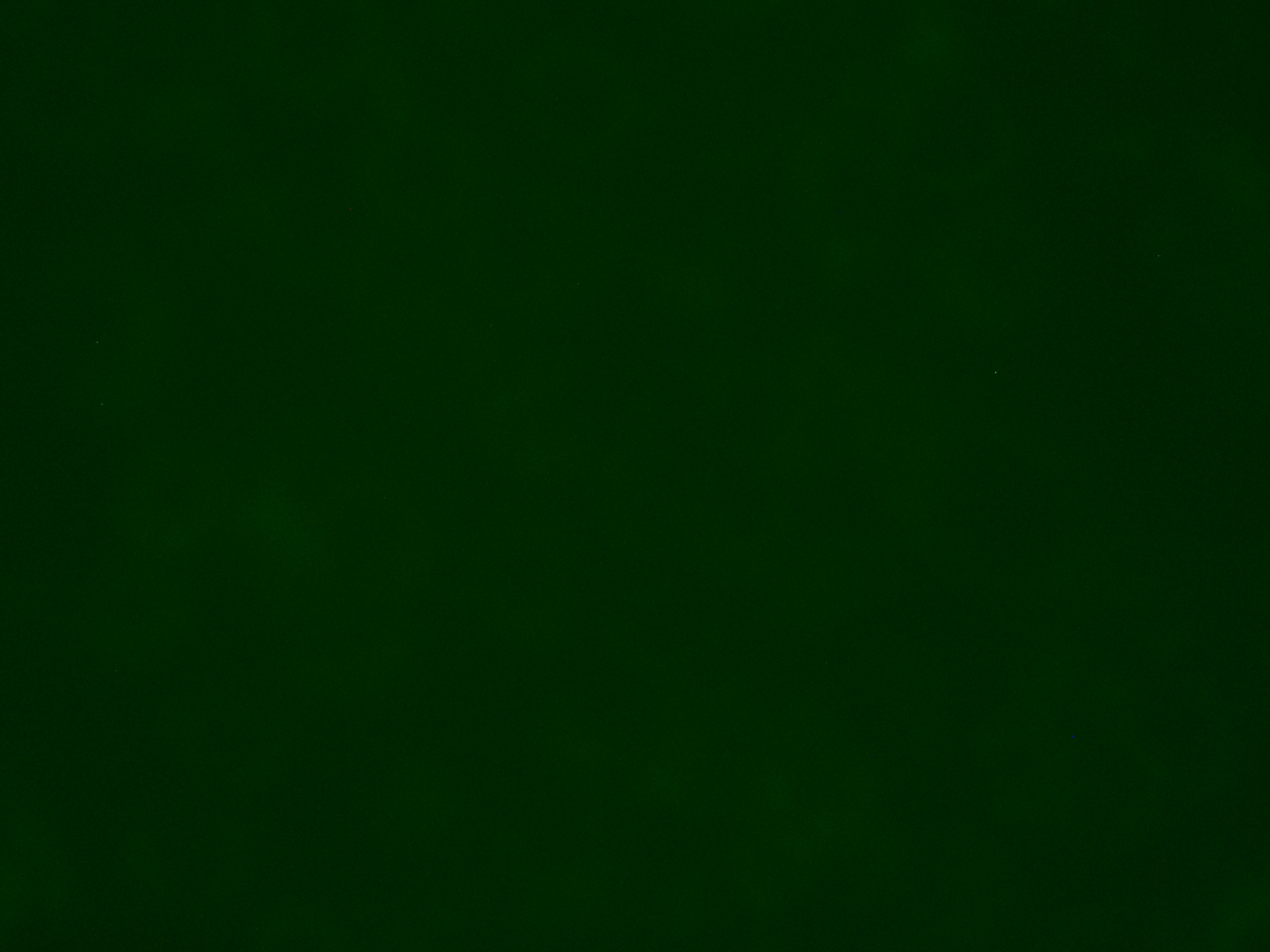

Supplement: S1 File — (ZIP) [file pone.0270734.s001.zip › S1 File/Figure 4A_PC3 cells exposed to Zn-S-NVC (conjugated to fluorescent dye) at 0 min/Fluorescence_Image.tif]

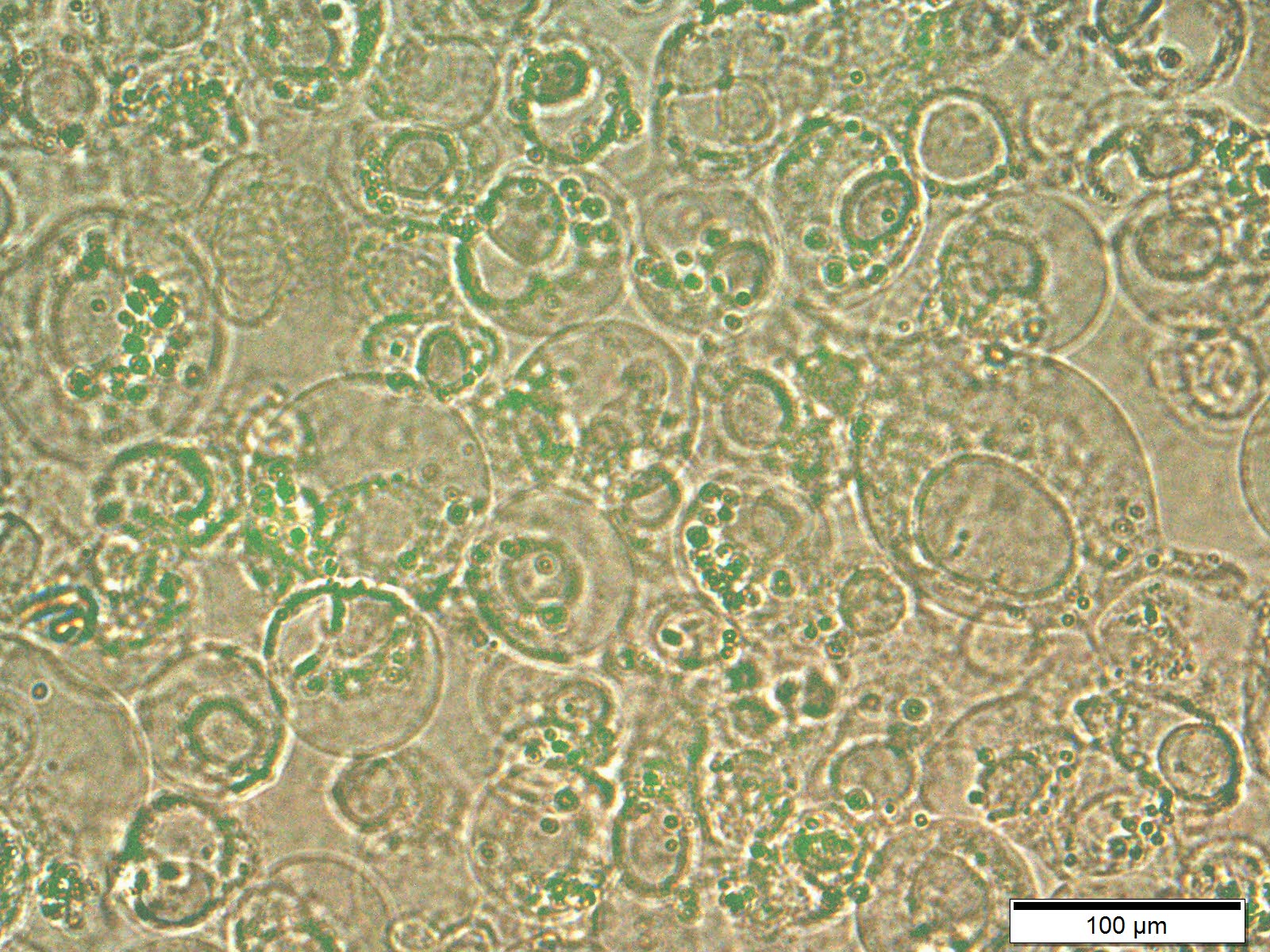

Supplement: S1 File — (ZIP) [file pone.0270734.s001.zip › S1 File/Figure 4A_PC3 cells exposed to Zn-S-NVC (conjugated to fluorescent dye) at 0 min/Merge_Brightfield_Image_and Fluorescence_Image.tiff]

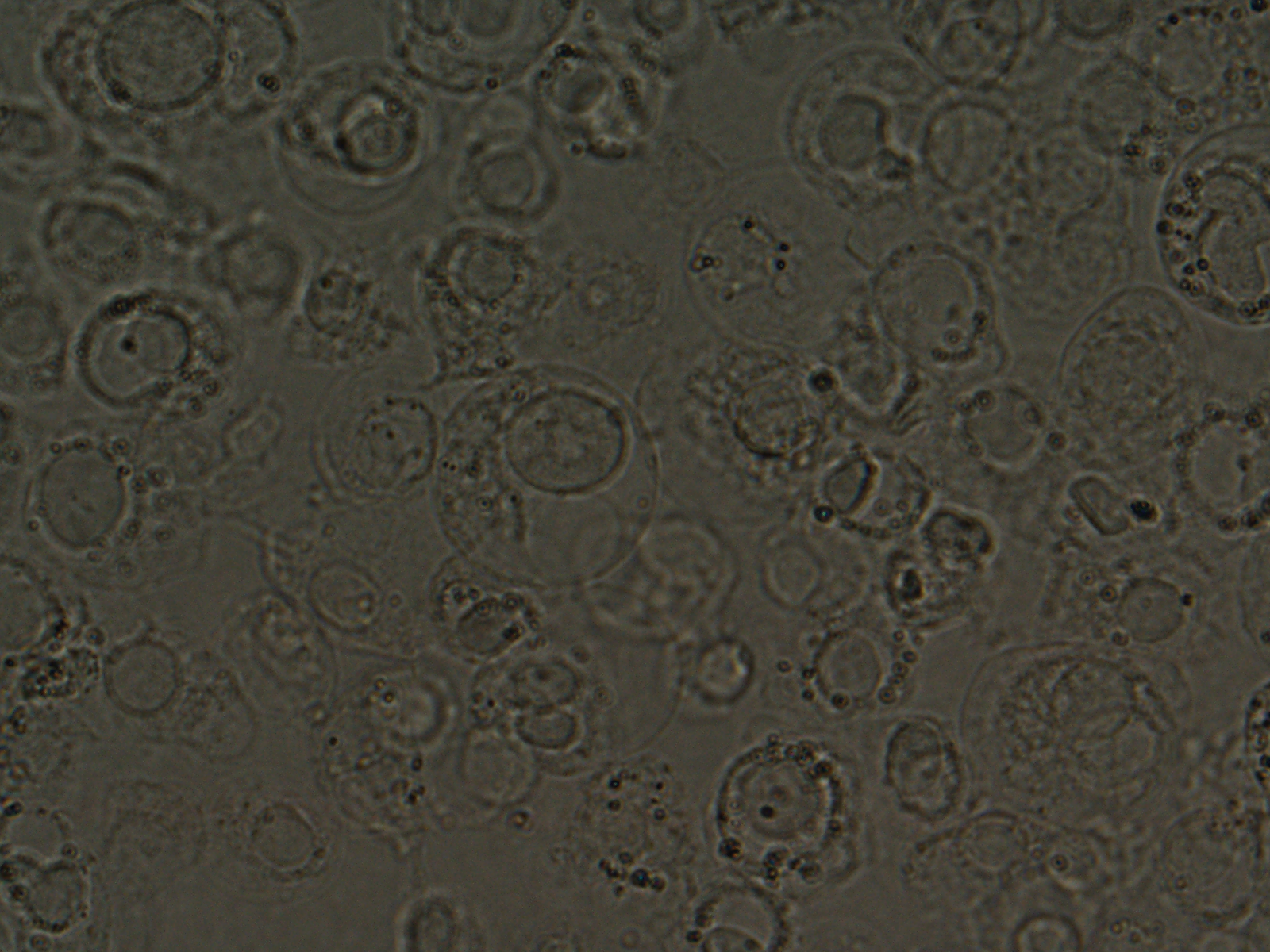

Supplement: S1 File — (ZIP) [file pone.0270734.s001.zip › S1 File/Figure 4B_PC3 cells exposed to Zn-S-NVC (conjugated to fluorescent dye) at 30 min/Brightfield_Image.tif]

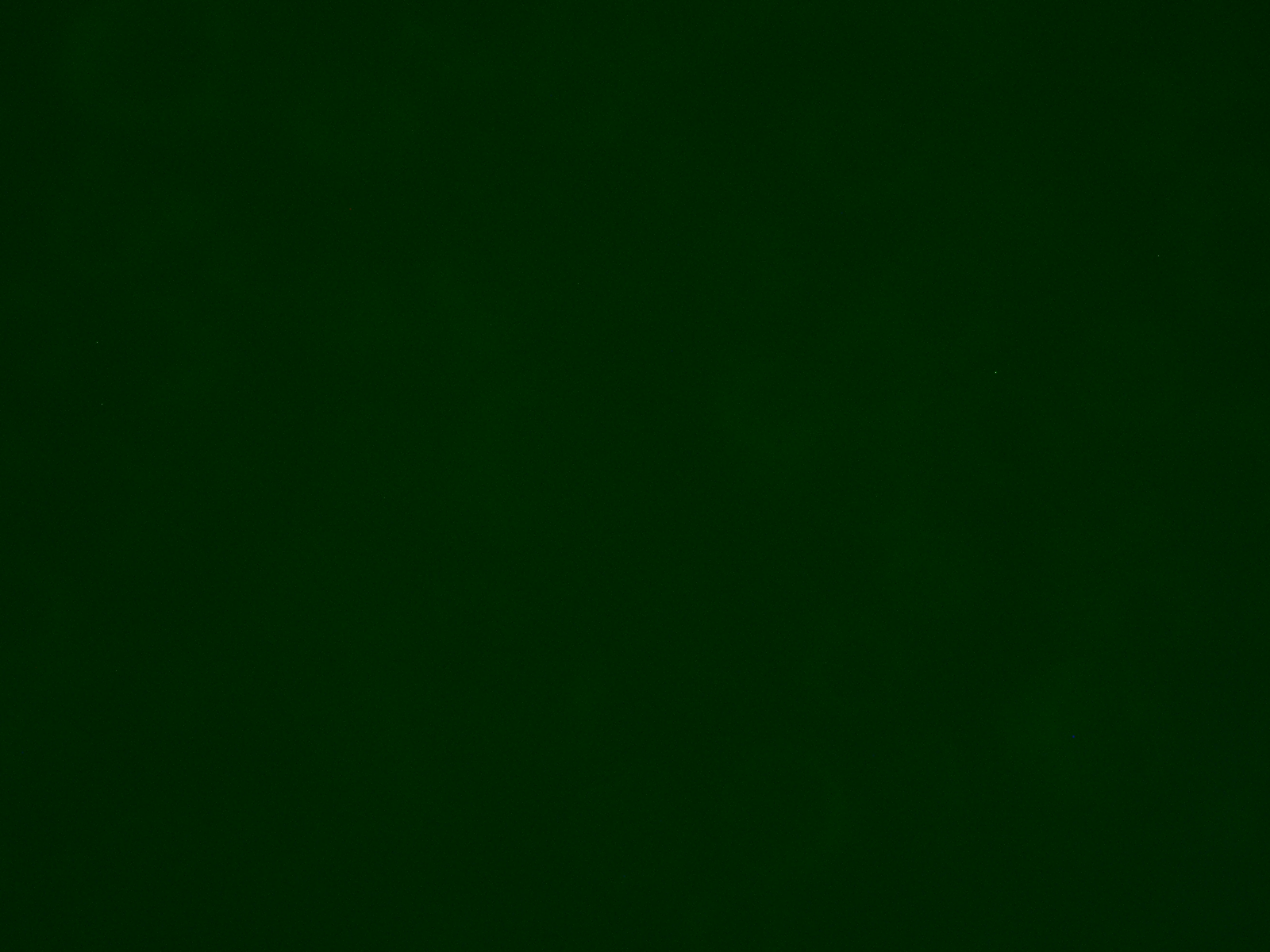

Supplement: S1 File — (ZIP) [file pone.0270734.s001.zip › S1 File/Figure 4B_PC3 cells exposed to Zn-S-NVC (conjugated to fluorescent dye) at 30 min/Fluorescence_Image_2.tif]

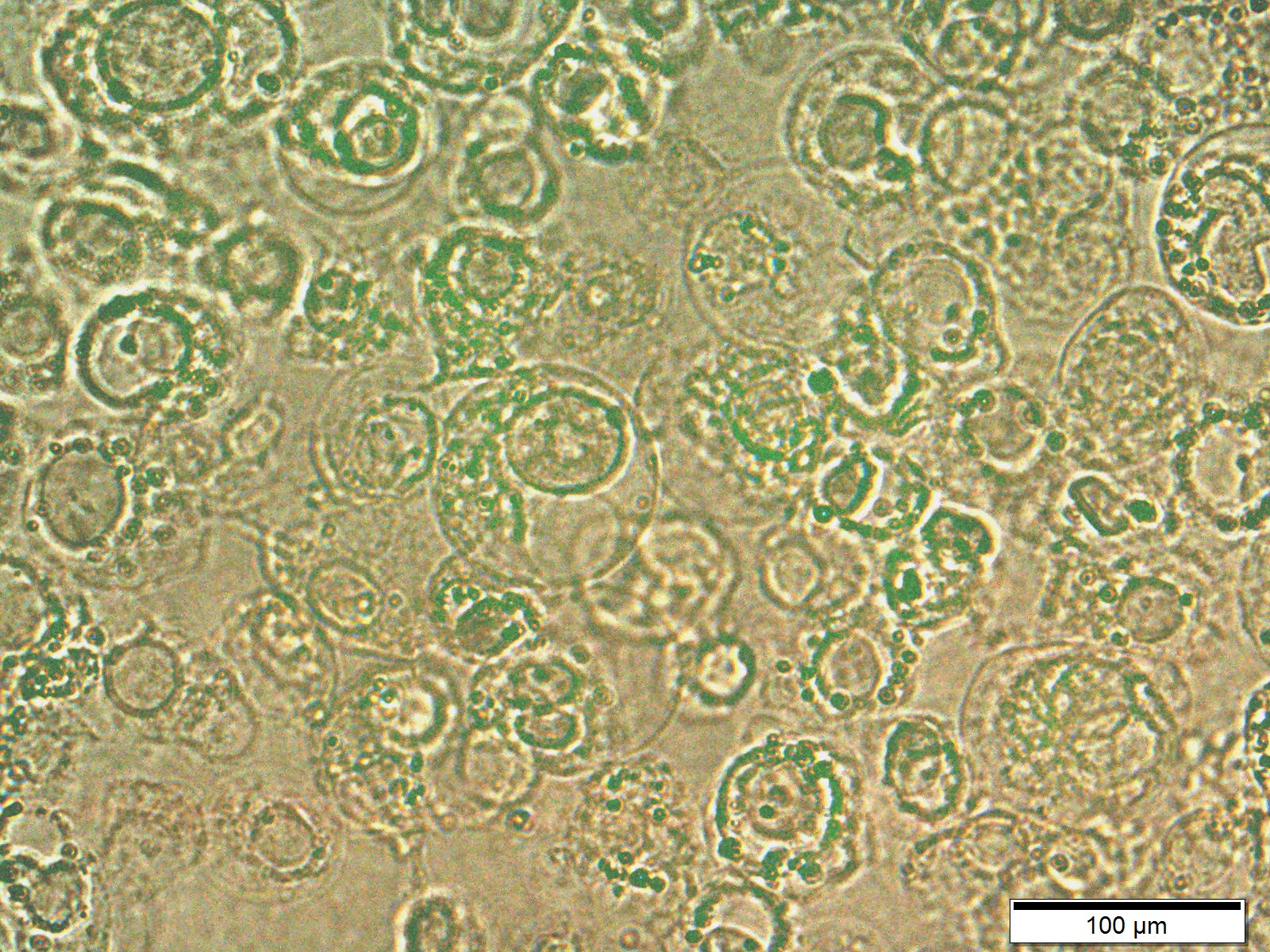

Supplement: S1 File — (ZIP) [file pone.0270734.s001.zip › S1 File/Figure 4B_PC3 cells exposed to Zn-S-NVC (conjugated to fluorescent dye) at 30 min/Merge_Brightfield_Image_and Fluorescence_image.tiff]

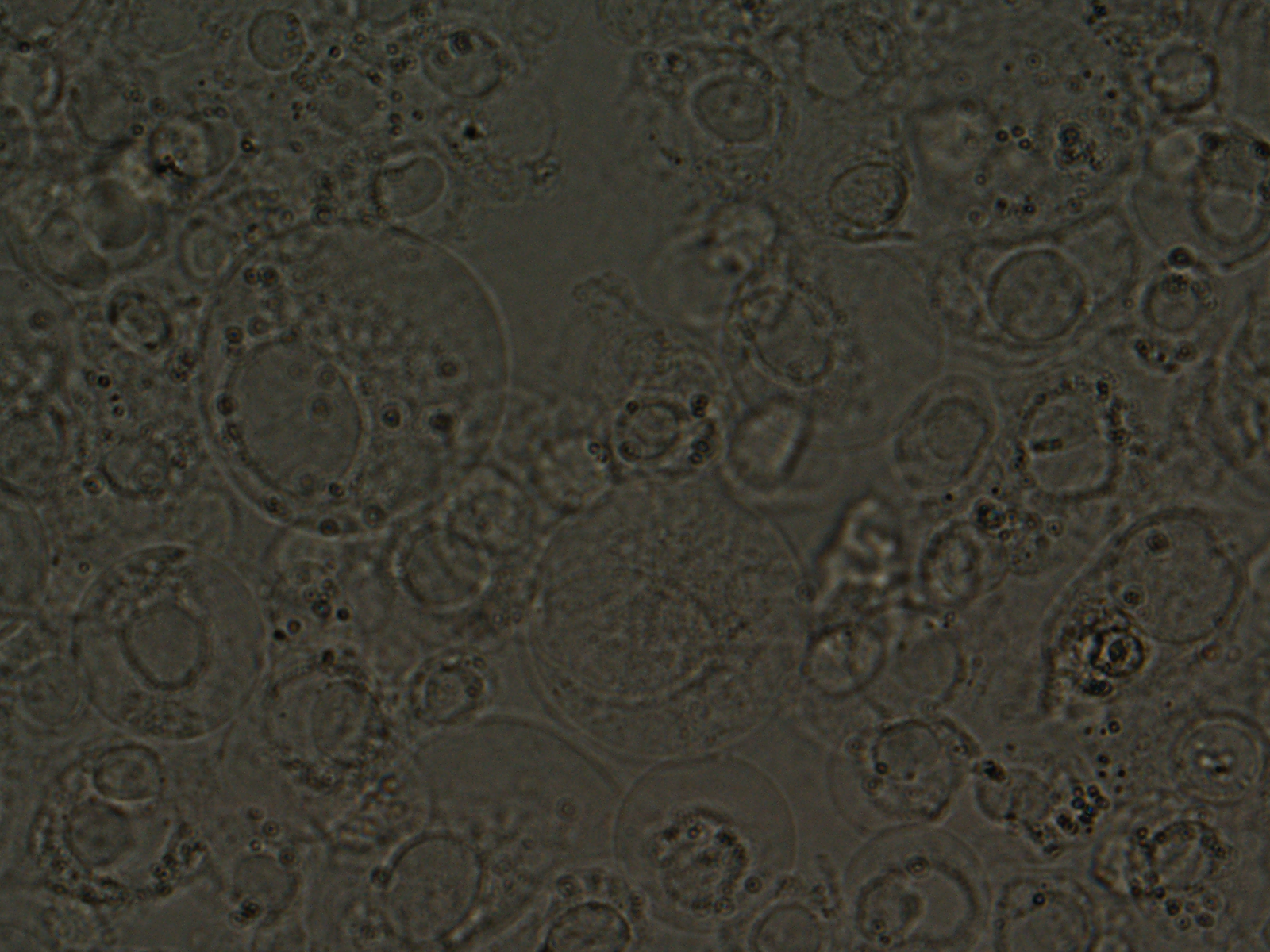

Supplement: S1 File — (ZIP) [file pone.0270734.s001.zip › S1 File/Figure 4C_PC3 cells exposed to Zn-S-NVC (conjugated to fluorescent dye) at 60 min/Brightfield_Image.tif]

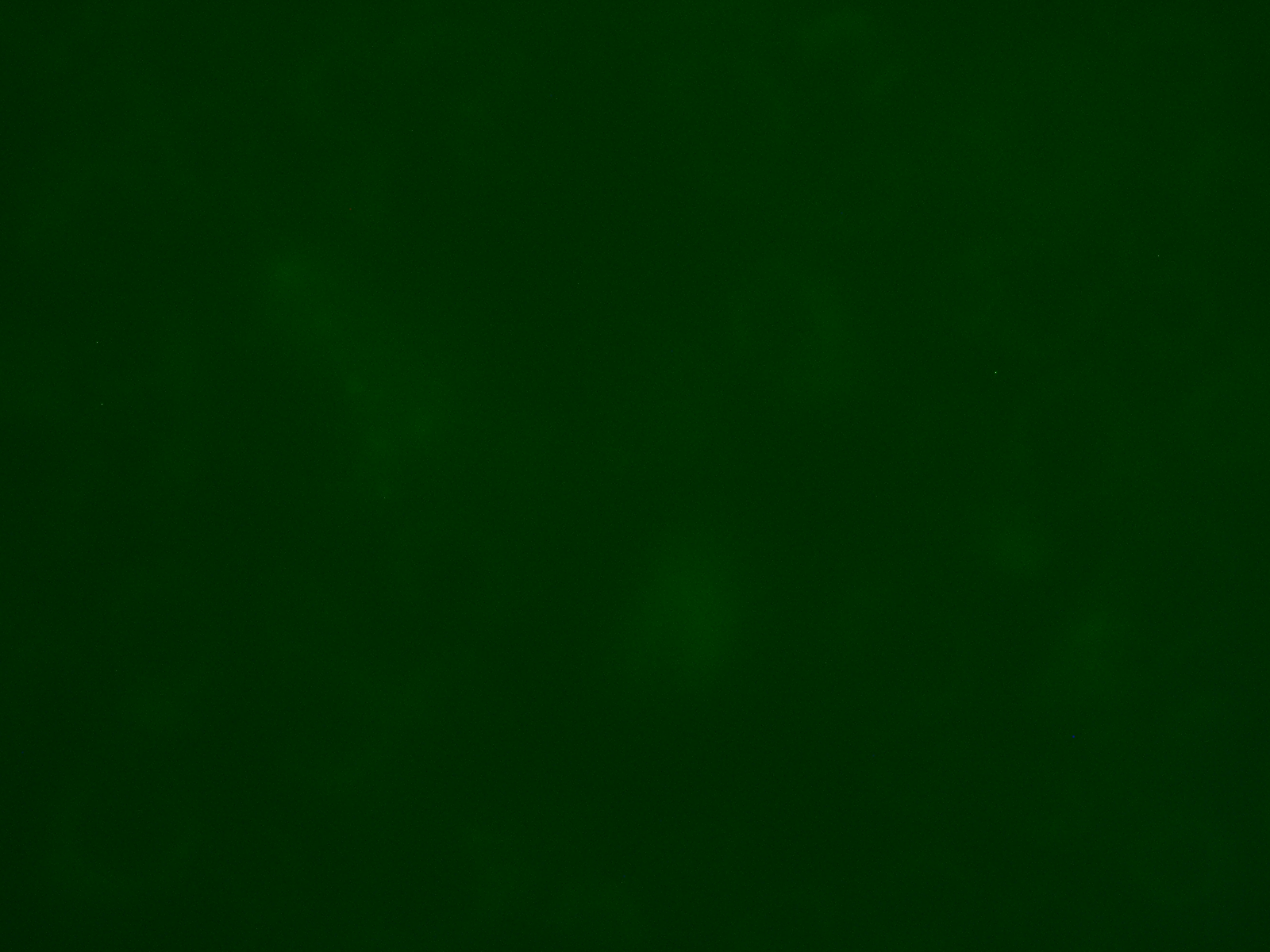

Supplement: S1 File — (ZIP) [file pone.0270734.s001.zip › S1 File/Figure 4C_PC3 cells exposed to Zn-S-NVC (conjugated to fluorescent dye) at 60 min/Fluorescence_Image.tif]

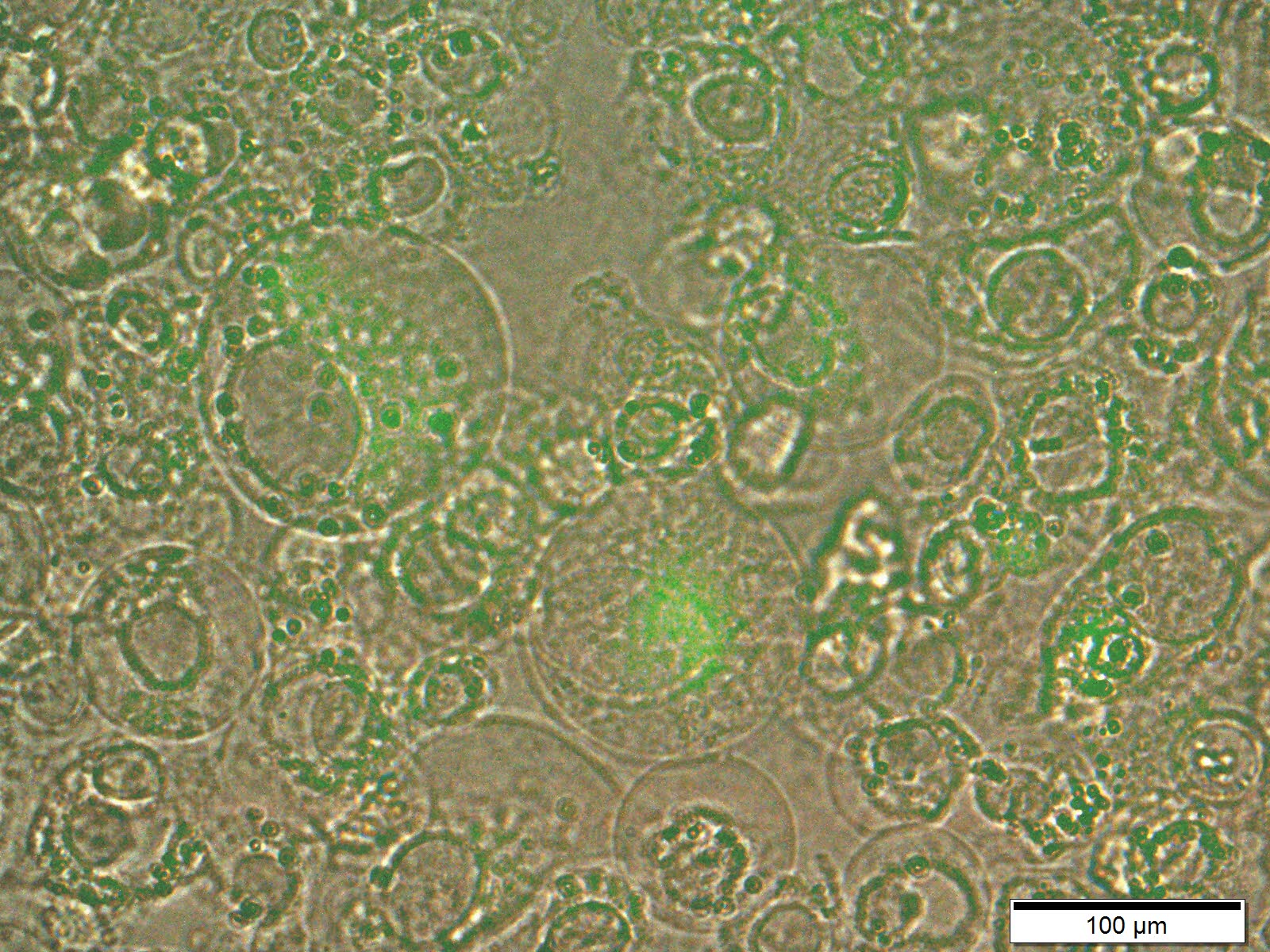

Supplement: S1 File — (ZIP) [file pone.0270734.s001.zip › S1 File/Figure 4C_PC3 cells exposed to Zn-S-NVC (conjugated to fluorescent dye) at 60 min/Merge_Brightfield_Image_and Fluorescence_Image.tiff]

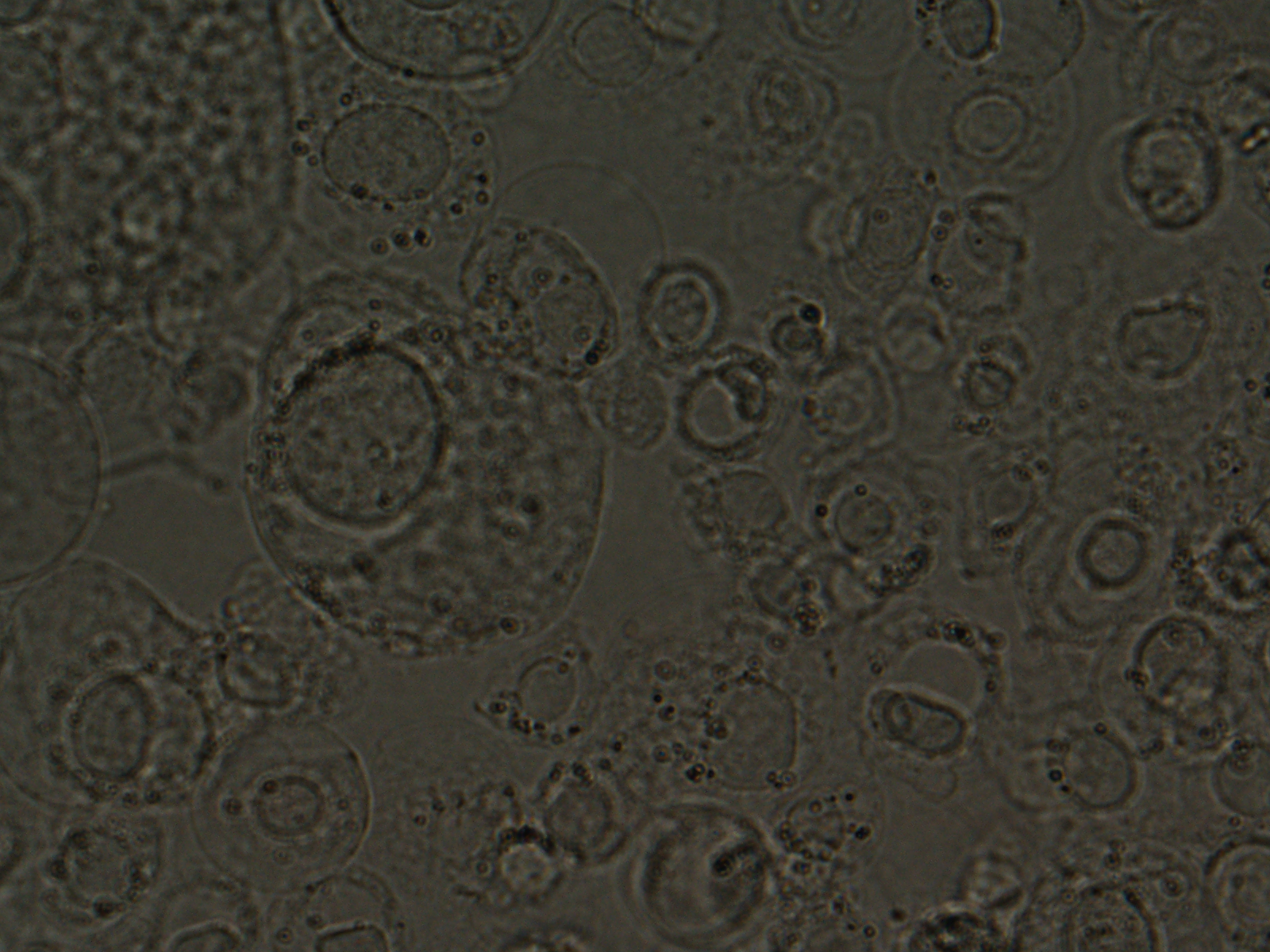

Supplement: S1 File — (ZIP) [file pone.0270734.s001.zip › S1 File/Figure 4D_PC3 cells exposed to Zn-S-NVC (conjugated to fluorescent dye) at 90 min/Brightfield_Image.tif]

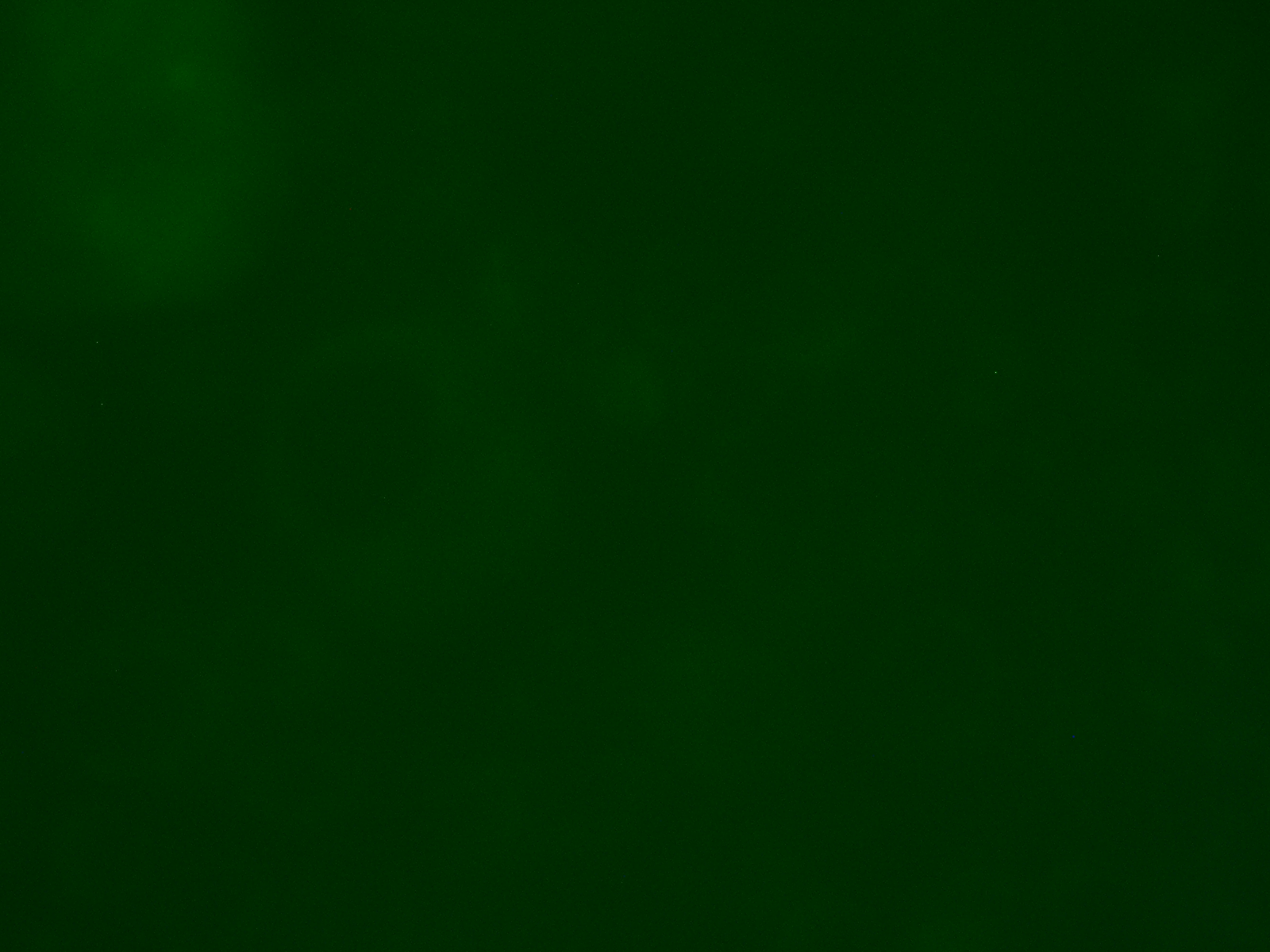

Supplement: S1 File — (ZIP) [file pone.0270734.s001.zip › S1 File/Figure 4D_PC3 cells exposed to Zn-S-NVC (conjugated to fluorescent dye) at 90 min/Fluorescence_Image.tif]

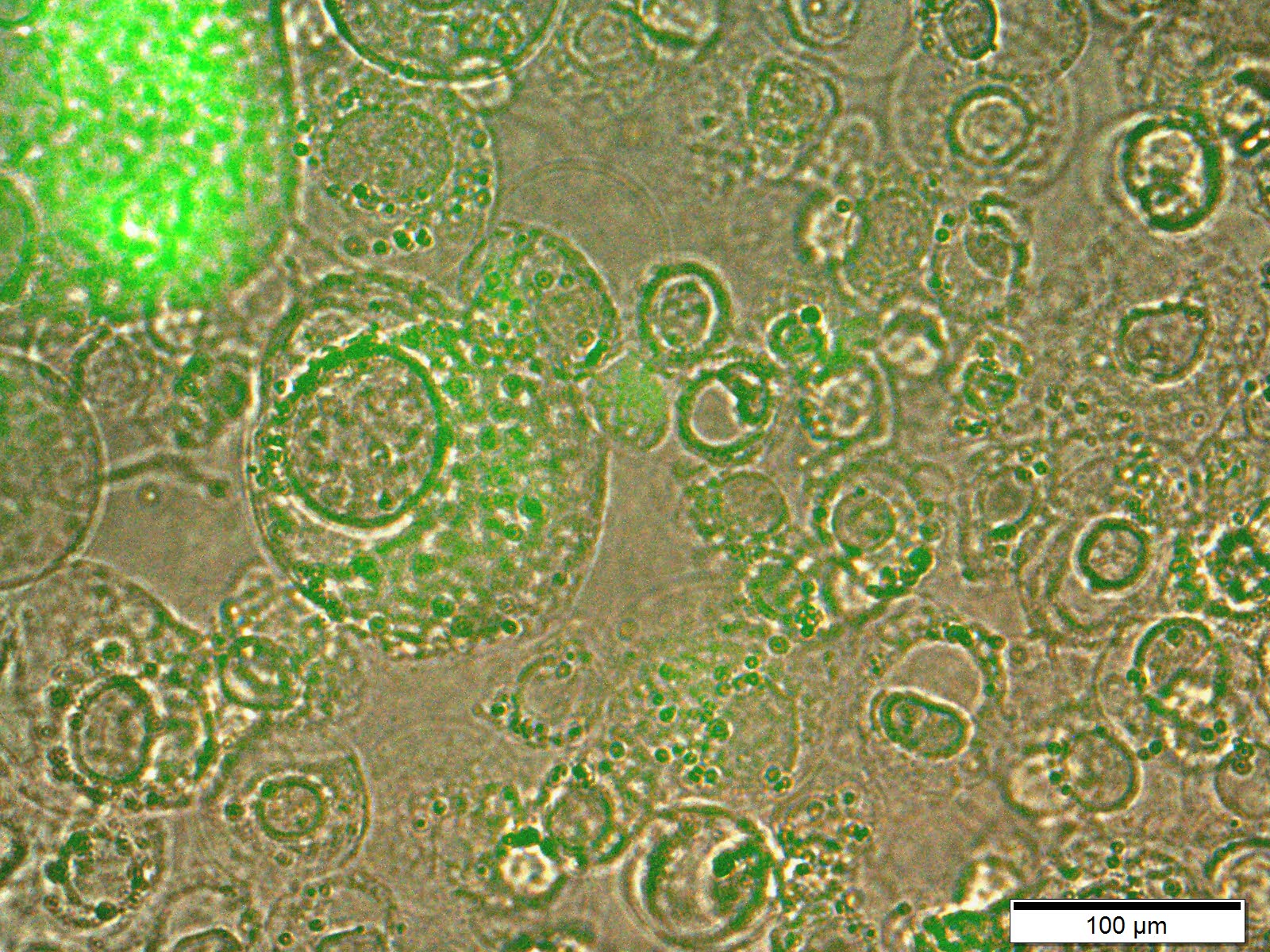

Supplement: S1 File — (ZIP) [file pone.0270734.s001.zip › S1 File/Figure 4D_PC3 cells exposed to Zn-S-NVC (conjugated to fluorescent dye) at 90 min/Merge_Brightfield_Image_and Fluorescence_image.tiff]

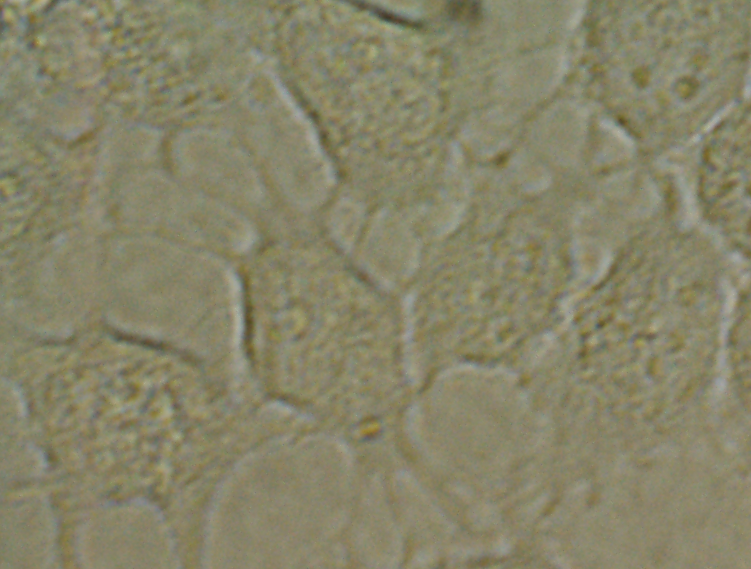

Supplement: S1 File — (ZIP) [file pone.0270734.s001.zip › S1 File/Figure 4E_PNT1A cells exposed to Zn-S-NVC (conjugated to fluorescent dye) at 0 min/Merge_Control_0min.tif]

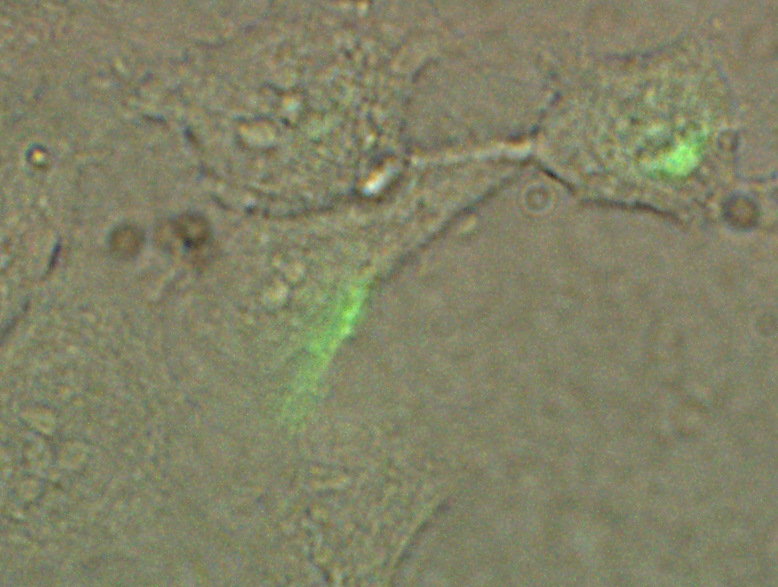

Supplement: S1 File — (ZIP) [file pone.0270734.s001.zip › S1 File/Figure 4F_PNT1A cells exposed to Zn-S-NVC (conjugated to fluorescent dye) at 30 min/Merge_30min_after_treatment.tif]

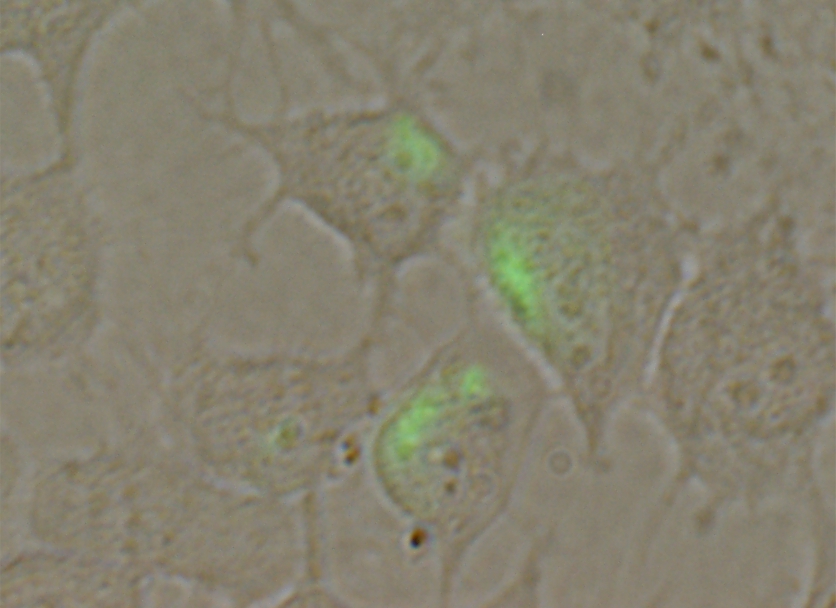

Supplement: S1 File — (ZIP) [file pone.0270734.s001.zip › S1 File/Figure 4G_PNT1A cells exposed to Zn-S-NVC (conjugated to fluorescent dye) at 60 min/Merge_60min_after_treatment.tif]

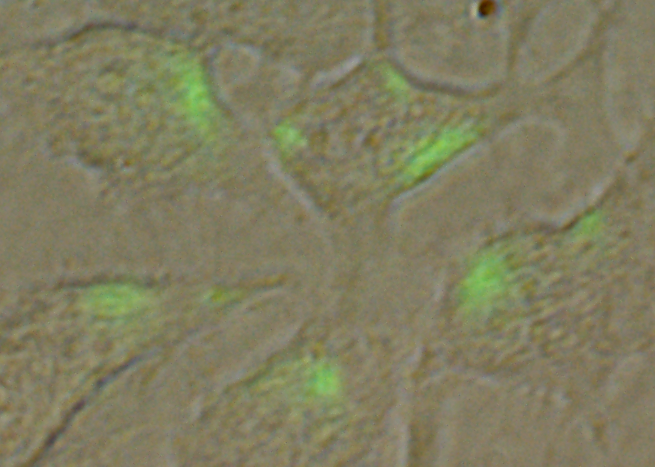

Supplement: S1 File — (ZIP) [file pone.0270734.s001.zip › S1 File/Figure 4H_PNT1A cells exposed to Zn-S-NVC (conjugated to fluorescent dye) at 90 min/Merge_90min_after_treatment.tif]
